# Operando Synchrotron-Based Fourier Transform Infrared Microspectroscopy of Metal-Ion Organic Battery Materials

**DOI:** 10.1021/acs.chemmater.5c01795

**Published:** 2026-01-07

**Authors:** Ashley P. Black, Deyana S. Tchitchekova, Nagaraj Patil, Nicolas Goujon, David Mecerreyes, Rebeca Marcilla, Ibraheem Yousef, Alexandre Ponrouch

**Affiliations:** a Institut de Ciència de Materials de Barcelona, ICMAB-CSIC, Campus UAB, Catalonia 08193, Spain; b ALISTORE − European Research Institute − CNRSFR 3104, Amiens 80039, France; c ALBA synchrotron, CELLS, Cerdanyola del Valles̀,Catalonia 08290, Spain; d Electrochemical Processes Unit, 202532 IMDEA Energy, Avda. Ramon de la Sagra 3, Mostoles 28935, Spain; e POLYMAT University of the Basque Country, Avda. Tolosa 72, Donostia-San Sebastian 20018, Spain; f Applied Chemistry Department, Faculty of Chemistry, University of the Basque Country EHU, San Sebastián 20018, Spain; g Ikerbasque, Basque Foundation for Science, 48013 Bilbao, Spain

## Abstract

*Operando* synchrotron-based Fourier transform infrared (SR-μFTIR) microspectroscopy takes advantage of the high brilliance of synchrotron radiation to collect high-quality spectra with a superior signal-to-noise ratio (S/N) in subsecond timeframes. This technique achieves a spatial resolution finer than ten micrometers, enabling real-time operando mapping of electrode surfaces during battery operation, even under high C rate. The combination of both high temporal and spatial resolution makes (SR-μFTIR) a powerful tool for investigating dynamic electrochemical processes at the microscale. *Operando* SR-μFTIR microspectroscopy can be applied to the study of electrode materials, electrolytes, and electrode–electrolyte interfaces. It is especially valuable for the elucidation of reaction mechanisms taking place in noncrystalline and/or lightweight element-based electrodes, such as organic electrode materials. Herein, we show how a simple modification of the commercially available ECC-Opto-Std (ELCELL) cell allows unraveling the potential of operando SR-μFTIR microspectroscopy for investigating organic electrodes. This setup is applied to study the reaction mechanism of polyimide derived from 1,4,5,8-naphthalenetetracarboxylic dianhydride (NTCDA) in lithium, sodium, and calcium cells. During the charge/discharge process of the polyimide, a reversible change in the carbonyl bands intensities is observed with the concomitant appearance of two main new bands. Density functional theory calculations assign these bands to competing enolation/carbonylation processes with direct interactions between the aromatic ring and alkaline metal ions present in the electrolyte. Furthermore, the enhanced spectral resolution of synchrotron radiation provides a more detailed insight into the stepwise mechanism pathway in Na cells, as well as rate-dependent variations in the reaction mechanism.

## Graphical abstract

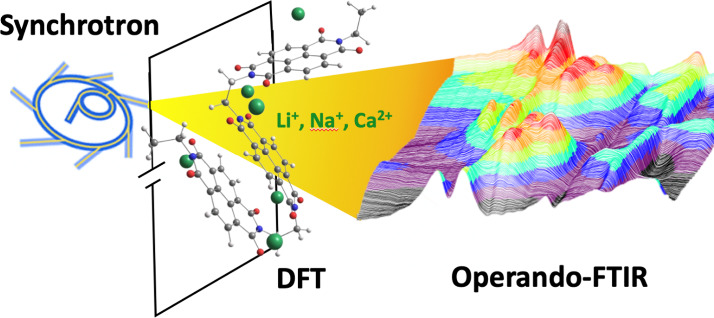

## Introduction

Fourier transform infrared (FTIR) spectroscopy is a powerful analytical technique that relies on the absorption of infrared light by molecules within a sample. This nondestructive method, characterized by photon energy less than 1 eV, is particularly effective for studying noncrystalline organic compounds. Absorption occurs when the frequency of incident IR light matches the vibrational frequency of a molecular bond, creating a unique molecular fingerprint that aids in the identification of the chemical components. In battery research, FTIR spectroscopy has become indispensable for chemical identification and structural determination, providing detailed insights into electrode redox mechanisms as well as electrolytes/solid electrolyte interphase composition and properties.^1–6^

*Operando* characterization of battery materials, conducted in real-time during battery operation, offers significant advantages. It enables the detection of transient metastable intermediates and ensures characterization under actual operational conditions, thereby avoiding artifacts associated with ex situ sample manipulation.^7^ This approach is crucial for elucidating redox mechanisms in emerging technologies and for understanding failure and aging processes in commercial battery systems. In situ and *Operando* FTIR measurements using conventional thermal sources have primarily focused on electrolyte degradation processes at the electrode–electrolyte interface, as well as on reaction mechanism in organic electrode materials.^4,8,17–21,17–21,9–16^

However, beyond the use of standard laboratory equipment, coupling FTIR with synchrotron radiation (SR-FTIR) offers significant advantages for studying battery materials. Synchrotron infrared light not only is spatially coherent but also possesses a photon flux that is two to three orders of magnitude greater than typical incoherent, thermal infrared sources. This exceptional brightness and coherence enable the probing of smaller regions (3–10 μm) with subsecond temporal resolution while maintaining an acceptable signal-to-noise ratio.^7,22^ Additionally, the broad spectral bandwidth of synchrotron sources allows full utilization of the entire infrared spectrum, from the far- to near-infrared regions. The pulsed time structure and high degree of polarization of synchrotron radiation further enable multimodal approaches, integrating techniques that require varying photon energies or frequencies. This capability enables comprehensive operando characterization beyond the limits of conventional laboratory-based infrared sources. Although ex situ SR-μFTIR has been employed to investigate the passivation layers in multivalent battery chemistries,^23^ operando FTIR measurements using synchrotron radiation have remained scarce, primarily due to the challenges associated with developing suitable electrochemical cells. Herein, we demonstrate, for the first time, that operando FTIR spectroscopy measurements using synchrotron radiation can be successfully applied to battery electrode materials using an ECC-Opto-Std (ELCELL) equipped with a 0.3 mm thick Si window. By leveraging the high temporal and spatial resolution of synchrotron radiation, we illustrate how *operando* microspectroscopy mapping of electrode surfaces can be conducted at high C-rates, providing unique insight into the elucidation of the redox reaction mechanism of a representative noncrystalline organic electrode material. Furthermore, we investigated the impact of different electrolytes containing various charge carriers. This is particularly relevant for organic electrodes, which exhibit exceptional versatility and redox activity across a wide range of charge carriers, making them promising candidates not only for monovalent chemistries such as Li and Na but also for emerging multivalent systems, including Mg- and Ca-based chemistries.^24–28^ Despite their potential, knowledge of the influence of different electrolytes on their electrochemical performances, particularly in terms of the redox potential and kinetic properties, is scarce. This study addresses this gap by providing new fundamental insights into the electrolyte-dependent redox behavior of organic electrodes, paving the way for their optimization in next-generation battery technologies.

## Materials and Methods

The cathode material 1,4,5,8-naphthalenetetracarboxylic dianhydride (NTCDA)-derived polyimide (PI) was synthesized by a polycondensation reaction between NTCDA and ethylenediamine as described previously.^29^ Self-standing buckypaper electrodes were prepared using single-wall carbon nanotubes (SWCNT) and reduced graphene oxide (RGO) (PI:SWCNT:RGO, 80:10:10 weight) as described previously.^30^

Operando Fourier Transform infrared spectroscopy (FTIR) was conducted at the MIRAS beamline of the ALBA synchrotron light source (Cerdanyola del Vallès, Spain), using a 3000 Hyperion microscope coupled to a Vertex 70 spectrometer (Bruker, Germany). The spectra were collected with a Mercury–Cadmium–Telluride (MCT) 50 μm detector using the internal source of radiation and the synchrotron light source. The microscope optics used a 36× Schwarzschild objective (NA = 0.52) to collect IR spectra in specular reflection configuration of the microscope in the mid-IR spectral range 700–4000 and 4 cm^–1^ spectral resolution with projected aperture sizes of 6 × 6, 10 × 10, 30 × 30, and 50 × 50 μm^2^ (depending on the single-point or raster scan (mapping) measurements). Each spectrum was collected by coadding 1, 5, 10, 30, 60, and 180 scans coupled to a scanner velocity of 40 kHz, which corresponds to 0.2 s per scan. To ensure spectral stability and a high signal-to-noise ratio during long operando SR-FTIR experiments, several methodological precautions were implemented. Beam stability was maintained using active steering and pre-/post-experiment monitoring at the synchrotron IR beamline, with background spectra acquired under identical optical conditions. Scattering effects were minimized by precise optical alignment and the selection of flat electrode regions through raster scanning. The high photon flux of the synchrotron source, combined with an optimized aperture size and scan number, enabled enhanced S/N performance over extended cycling periods. Repeated measurements on multiple cells confirmed the reproducibility and reliability of the observed spectral evolution.

Operando SR-FTIR measurements were conducted in the specular reflection mode using a synchrotron IR beam. In this configuration, the incident IR radiation reflects at a fixed angle from the electrode/electrolyte interface, and the reflected intensity carries absorption features arising from surface species (e.g., interphase or decomposition products). The measurements were performed in an ECC-Opto-Std (ELCELL) instrument equipped with a 0.3 mm thickness Si window. Electrochemical tests were performed on the self-standing PI ((80:10:10), (PI:SWCNT:RGO))^30^ electrodes employing 1 M LiTFSI (99%, Aldrich), 1 M LiPF_6_ (99.9%, Aldrich), 1 M NaTFSI (99.9%, Aldrich), 0.5 M Ca­(TFSI)_2_ (99.5%, Aldrich) or 0.5 M Ca­(BF_4_)_2_ (Alfa Aesar) in an ethylene carbonate (EC, anhydrous 99.0%, Aldrich) and propylene carbonate (PC, anhydrous 99.7%, Aldrich) mix 1:1 wt %, or in diglyme (DG, anhydrous, 99.5%, Aldrich). The water content in the electrolytes was measured by Karl Fischer titration and found to be lower than 20 ppm in all cases. Electrolyte preparation and cell assembly were always carried out inside an argon-filled glovebox with <1 ppm of H_2_O and O_2_. Counter electrodes were made from activated carbon cloth (AC) (Kynol, ACC-509220) dried under a vacuum at 75 °C overnight prior to use.^31^ The number of disks and diameter (2–3, ⌀ 8–10 mm) were adjusted to the working electrode mass to ensure proper capacity balancing, with 3 times excess capacity of AC with respect to the working electrode active material. Cells were cycled in galvanostatic cycling with potential limitation (GCPL) mode between C/4 and 2.5C rates using a Bio-Logic SP-200 potentiostat with 1C being 184 mA g^–1^, considering the molar mass of the naphthalene diimide unit (292 g/mol) and the number of electrons typically exchanged through coordination with Li^+^ (here, *n* = 2).

Initially, 12 conformations for the neutral NTCDI and 12 isomers for each of the bicoordinated complexes 2Li-NTCDI and 2Na-NTCDI were created in Avogadro.^32^ DFT optimizations of all geometries were performed with the ORCA 5.0.3 package^33^ using the B3LYP functional with Grimme’s D3 empirical dispersion correction and def2-TZVPP basis set.^34^ The optimized geometries were confirmed as energy minima from the computed vibrational frequencies at the same level of theory. Relative total energies with respect to the minimum energy structure were calculated as Δ*E*_rel_ = *E*_isomer_ – *E*_min_, accounting for zero-point energy corrections. Gibbs free energies for the interaction between the neutral NTCDI and two Li or two Na atoms were obtained at 298.15 K as Δ*G*_rel_ = *G*_isomer_ – *G*_NTCDI_ – 2 × *G*_Li/Na_. Additional calculations were carried out for monocoordinated Li–NTCDI and Na–NTCDI complexes at the same level of theory. Enthalpies of formation (Δ*H*_*f*_) were calculated for the most stable mono- and bicoordinated isomers of each system, and incremental enthalpies for the binding of the second cation as ΔΔ*H*_*b*,2_ = Δ*H*_*f*2_ – Δ*H*_*f*1_.

## Results and Discussion

In this study, we selected polyimide-derived 1,4,5,8-naphthalenetetracarboxylic dianhydride (NTCDA) that has demonstrated good electrochemical performance in both monovalent (Li^+^ and Na^+^) and divalent (Ca^2+^ and Mg^2+^) batteries (Figure S1).^30^ While FTIR is particularly well-suited for tracking the carbonyl and imide stretching modes that dominate the redox activity of NTCDA-derived polyimides, operando Raman spectroscopy could offer complementary vibrational information, especially from the aromatic core of the polyimide structure. However, its application is often limited by fluorescence and weak signal intensity in carbon-rich or conjugated materials.

In our previous work, operando FTIR spectroscopy using a Globar source was employed to observe the evolution of all infrared-active bands and assess the reversible mechanism of the redox-active carbonyl groups in the imide functionality of PI via enolation/carbonylation reactions.^30^ However, in this study, we leverage the superior brilliance of the synchrotron infrared source, which offers significantly higher spatial and temporal resolution compared with conventional thermal sources, while maintaining an optimal signal-to-noise ratio. This advancement enables a more detailed and precise analysis of the reaction mechanisms at the electrode surface, providing deeper insights into the electrochemical processes at play.

### SR-FTIR Windows

When transitioning from the internal thermal source of the spectrometer to synchrotron radiation, some of the typical IR transparent windows in the mid-IR frequency range (CaF_2_, SrF_2_, ZnSe)^9,10,20,21^ showed a magnified interference pattern. To identify a suitable window for synchrotron radiation, several window materials were tested. Figure 1 displays the spectrum of the PI electrode measured with synchrotron radiation, both directly under the microscope and within the ELCELL using windows of various compositions and thicknesses.

**Figure 1:**
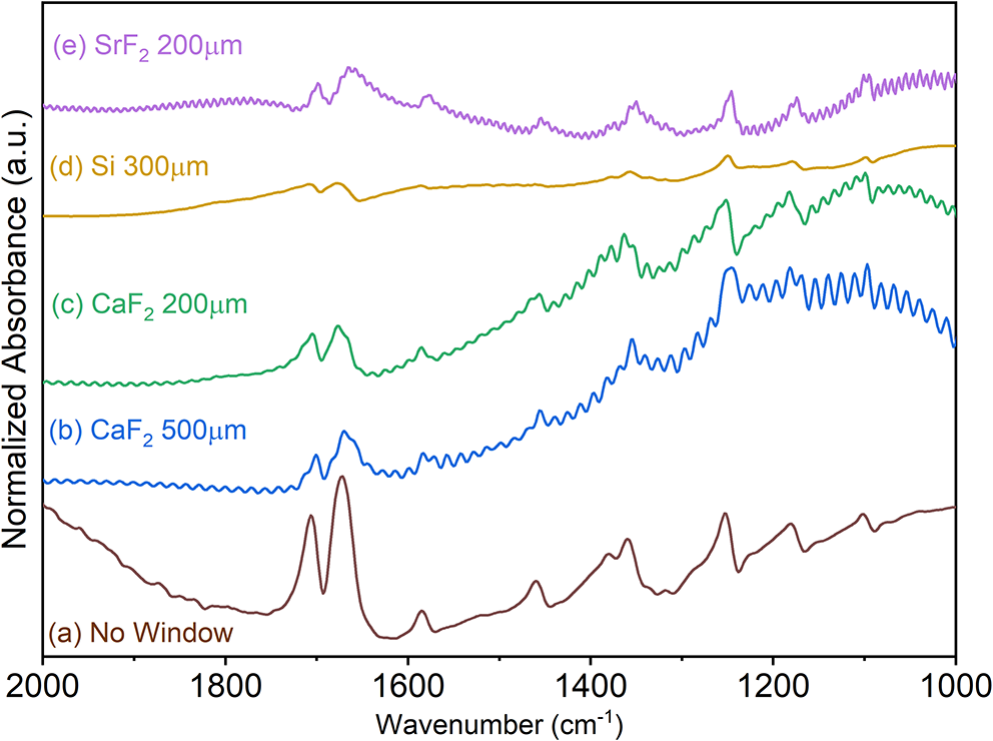
IR spectra in the region 2000–1000 cm^–1^ of a dry PI electrode directly under the IR microscope with no window (a) in the ECC-Opto-Std (ELCELL) with a 500 μm CaF_2_ (b), 200 μm CaF_2_ (c), 300 μm Si (d), or a 200 μm SrF_2_ (e) window.

The spectra measured with CaF_2_ and SrF_2_ exhibited strong interference patterns across the entire spectral range. Additionally, ZnSe windows (not shown) also presented strong interference patterns. These patterns result from the partial reflection of light at the interfaces between materials with different refractive indices. CaF_2_ and SrF_2_ have refractive indices of approximately 1.4 and 1.44, respectively, while ZnSe has a higher refractive index of about 2.4. This interaction can cause constructive or destructive interference, depending on the wavelength of the radiation, the refractive indices of the materials, the thickness of the layer, and the angle of the incident radiation (see Figure S2). Higher refractive indices generally lead to stronger reflections at interfaces, since greater refractive indices result in higher reflection coefficients, increasing the likelihood of interference patterns. For CaF_2_, the frequency of interference was found to be 13 cm^–1^ for both the 200 and 500 μm-thick windows, while SrF_2_ showed a higher interference frequency of 8 cm^–1^ (see Figure S3A). Tests conducted with and without an electrolyte allowed for ruling out interference caused by an electrolyte layer between the window and the sample. Using the internal thermal source (Globar) of the Vertex spectrometer, the interference patterns observed with the CaF_2_ windows were still present but had a significantly smaller amplitude, almost blending into the noise (see Figure S3B).

Among the tested IR transparent windows, silicon was one of the materials that did not exhibit interference patterns in the spectral signal when the synchrotron source was used. Despite having a high refractive index of around 3.4 in the infrared region, which would typically result in significant partial reflections, silicon’s unique dispersion properties can help spread out any potential interference effects over a broader spectral range, reducing the visibility of distinct interference fringes. Additionally, although the silicon lets most of the mid-infrared light pass right through (the energy level carried by the IR light photons being too low compared with the material’s bandgap, 0.5 to 0.05 eV vs. 1.1 eV), it still possesses some absorption through phonon interactions and free-carrier absorption in the mid-infrared region attenuating the intensity of the reflected light within the material. Therefore, interference patterns are minimized, leading to improved spectrum quality. This stronger dispersive capability of Si results in the partial reflection from the lower part of the window being significantly attenuated within the thickness of the window (300-μm). Apart from the type of IR window material, there are additional factors that may contribute to this interference artifact, such as the window’s thickness, the spectral resolution selected, and the frequency range of the source.^35^

### Synchrotron Radiation vs Thermal Source

The superior brilliance of the synchrotron infrared source compared with the conventional thermal source allows for higher spatial and temporal resolution without sacrificing the signal-to-noise ratio. To compare the spatial and temporal resolution limits of the synchrotron source with those of a conventional thermal source, a series of spectra was measured with a beam spot size of 30 × 30, 10 × 10, and 6 × 6 μm^2^, accumulating 1, 5, 10, 30, 60, and 180 scans, where each scan took 0.2 s.

From the spectra depicted in Figure 2, we observe that our experimental setup achieves comparable signal-to-noise ratios with both sources for a beam spot size of 30 × 30 μm^2^ when measured for 1 scan with the synchrotron or 60 scans with the Globar source. However, at 10 × 10 μm^2^, the Globar source could not provide a satisfactory spectrum even after accumulating 180 scans, while the synchrotron source required only 5 scans to produce a quality spectrum at this spatial resolution. Exploring the instrumental limits within our experimental setup, we found that the highly collimated synchrotron source could measure with a satisfactory signal-to-noise ratio for a field of view of 6 × 6 μm^2^ with an acquisition time corresponding to 30–60 scans. Detailed sequential comparisons of different acquisition conditions are available in the Supporting Information (Figure S4). Under these experimental conditions, the use of synchrotron radiation allows for monitoring chemical transformations with subsecond temporal resolution and a spatial resolution higher than 10 μm.

**Figure 2:**
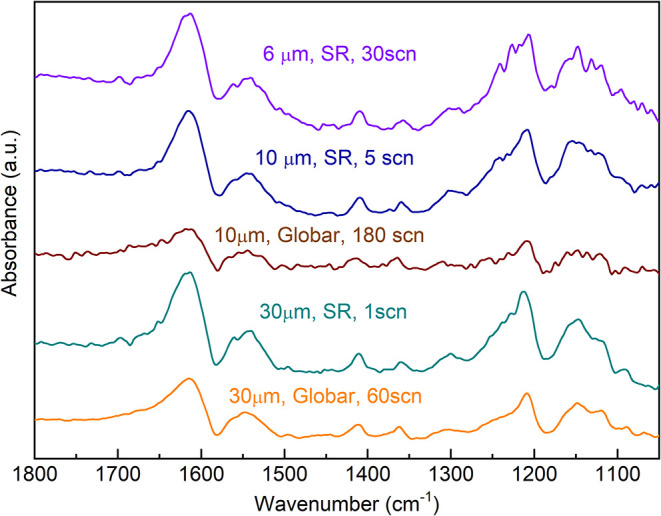
IR spectra in the region 1800–1050 cm^–1^ of PI electrode in the ECC-Opto-Std (ELCELL) with 1 M LiPF_6_ in DG electrolyte with 300 μm Si window measured with the Globar thermal source or the synchrotron radiation at 30 × 30, 10 × 10 and 6 × 6 μm^2^ (aperture size) for different number of scans. Exemplifying the limits of time and space resolution under these experimental conditions.

### SR-FTIR Mapping

Many studies on battery materials using in situ/*operando* ATR-FTIR focus on single-point measurements, typically around 100 × 100 μm^2^ or more. This approach can be problematic due to the difficulties in determining whether the measured point is representative of the entire electrode, leading to potentially misleading conclusions. To address this issue, it is essential to conduct multiple or raster scan measurements to verify the chemical composition across the electrodes. The FTIR technique’s ability to distinguish between different molecules allows for the visualization of chemical variations throughout the sample. Thus, utilizing synchrotron-based FTIR raster scanning can more effectively determine whether sample variations are heterogeneous or localized hotspots, enhancing our understanding of the electrode’s chemical composition.

Combining the subsecond acquisition rate at smaller light spot sizes below 30 × 30 μm^2^ with a motorized stage allows for mapping the chemical composition of a large area of the sample in just a few minutes with high spatial resolution.

Figure 3 shows a microspectroscopic FTIR map of 1400 × 1200 μm^2^, measured with a spectral resolution of 50 × 50 μm^2^, completed in 1 h. The chemical imaging in Figure 3 corresponds to the integrated intensity between 1700 and 1500 cm^–1^, associated with the carbonyl groups. The resulting map reveals an inhomogeneous chemical distribution of these bands, with some regions displaying much higher intensity compared to others. This uneven distribution is believed to be related to the depth distribution of active material inside the self-standing electrode and/or a different degree of separation between the electrode and the Si window. Indeed, the depth sensitivity of the incident light is limited and penetrates the cell (below the window) in the first 1–3 μm, with the electrode thickness being higher than 10 μm. The dark blue regions of the map correspond to areas of the electrode with an inverted infrared intensity of the corresponding C=O bands (Figure S5). This inversion is due to the nature of the interaction of light with the material: bands that appear with positive intensity correspond to light absorption, while inverted (downward) bands indicate interaction in the specular reflection mode. It appears that the interaction is primarily absorptive; however, some regions of the electrode, potentially related to the distance from the window, interact with the light in the reflection mode. The SR-FTIR mapping of the electrode allows for the localization and selection of optimal sample spots exhibiting the highest intensity for conducting operando measurements.

**Figure 3:**
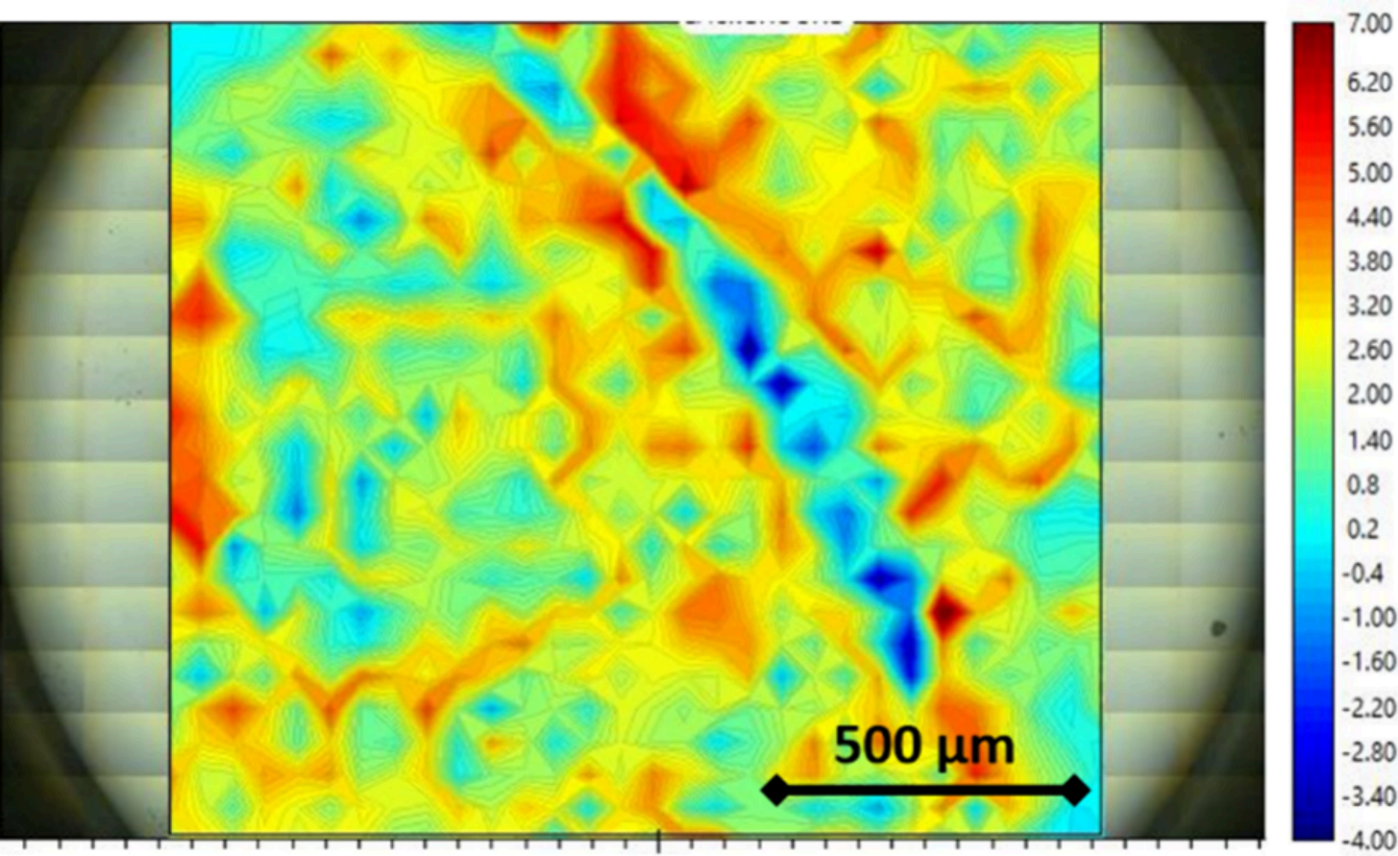
Large area (1400 × 1200 μm) microspectroscopic SR-FTIR map of the PI electrode in the ECC-Opto-Std (ELCELL) with 1 M Ca­(TFSI)_2_ in DG electrolyte with a 300 μm Si window showing the integrated intensity of the IR active bands present in the 1700–1500 region. Red area corresponds to high-intensity regions “hot spots” and blue areas correspond to inverted bands (look downward) where the light interacts in reflection with the sample.

In a previous work, we used the same electrochemical cell setup with a CaF_2_ window instead of a Si window and the same electrode material, but with a carbonate-based electrolyte (EC:PC) instead of glyme (G2).^30^ When studying organic electrode materials, selecting an appropriate electrolyte is crucial, as active bands of the material might potentially overlap with those of the solvent or salt, such as the carbonyl bands of the carbonate solvents and those of the organic cathode. We found that glyme-based solvents are a suitable alternative to carbonate-based solvents, offering a wide range of the infrared spectrum with few or no active bands, minimizing the risk of bands overlapping with those of the active material. The CaF_2_ window did not allow for synchrotron radiation measurements due to strong interference patterns that masked the signal (Figure S3B), so the operando experiments were conducted using a Globar source. Despite these differences, limiting the comparability of the results, data acquired with synchrotron radiation showed better band shape definition, allowing for finer spectral details and providing more accurate insight into the reaction mechanism (Figures S15 and S16).

### Operando SR-FTIR and DFT Modeling

Operando SR-FTIR spectroscopy allows real-time monitoring of the changes in the infrared active bands during electrochemical charge and discharge cycles, providing valuable insights into the redox mechanisms occurring within the electrode. In PI electrodes, the reversible redox mechanism involves enolation/carbonylation reactions of the redox-active carbonyl groups, accompanied by the reversible coordination/decoordination of cations during the discharge (reduction) and charge (oxidation) processes. Figure S6 in the Supporting Information presents the SR-FTIR spectra of the pristine PTCDA electrode (A), the electrolyte solution (1 M NaTFSI in diglyme, B), and the assembled cell containing the PTCDA electrode and 100 μm of electrolyte (C), together with the corresponding assignments of their main vibrational bands. Notably, no characteristic bands attributable to SWCNT or RGO are discernible in the spectrum of the pristine electrode (A), and the electrolyte signals in the assembled cell (C) are very weak, if detectable at all.

Figure 4 and Figure S7 display the operando SR-FTIR spectra of PI electrodes during electrochemical charge/discharge cycles in lithium-, sodium-, and calcium-based organic electrolytes. The PI electrodes achieved reversible capacities of 139–129 mAh/g in Li-based cells, 159–152 mAh/g in Na-based cells, and around 60 mAh/g in Ca-ion cells. The overall spectral evolution in both the Li and Na systems (Figure 4) follows similar trends, suggesting that the reaction mechanisms are comparable in both cases.

**Figure 4:**
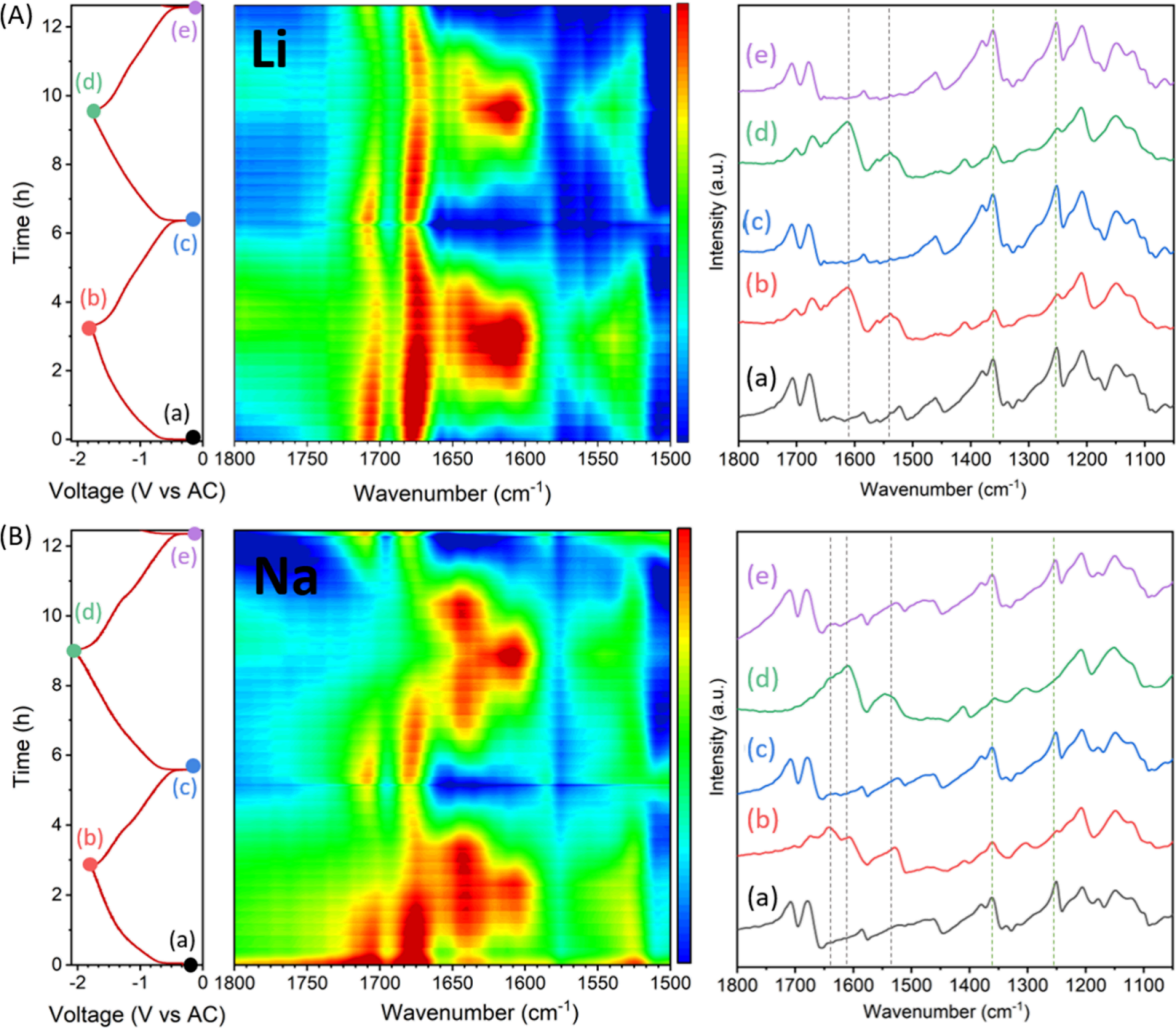
GCPL profile of PI at C/4 in LiTFSI in DG (A) and in NaTFSI in DG (B), their corresponding contour plot of the operando SR-FTIR spectra in the region 1800–1500 cm^–1^ and the selected spectra corresponding to the initial state (a), end of first reduction (b), first oxidation (c), second reduction (d), and second oxidation (e).

More specifically, upon reduction, the intensities of the carbonyl bands at 1703 and 1673 cm^–1^ progressively decrease, eventually disappearing at deep discharge down to −2.1 V vs AC (Figure 4B­(b,d)) for Na-based cells. For Li-based cells (Figure 4A­(b,d)), a significant decrease in the intensity of the carbonyl bands is observed upon deep discharge, yet not fully disappearing, and experiencing a slight red shift. Similarly, the bands at 1355 and 1251 cm^–1^, corresponding to the (C–N) vibration of the imide and the breathing mode of the naphthalene ring, also decreased in intensity. Concomitantly, two new intense broad bands emerge at 1630 and 1540 cm^–1^, along with two lower-intensity bands at 1407 and 1304 cm^–1^. In the case of Ca cells, the corresponding spectral changes are proportionally smaller (Figures S7A and S8). Nevertheless, the same new bands at 1620 and 1540 cm^–1^ appear.

The assignment of the experimental IR bands was accomplished by comparison with the theoretical spectra, presented in Figure 5A (for pristine NTCDI) and Figure 5B (for M-NTCDI, where M = Li^+^, Na^+^). In our calculations, we used one monomeric unit terminated with −CH_2_–CH_3_ groups to model the polymeric structure at a reasonable computational cost. The −CH_3_ and −CH_2_– stretching modes fall outside the analyzed frequency range, and the −CH_3_ deformations appear as weak intensity bands and are not considered in the overall analysis. The computed frequencies, detailed in Table S1 (for pristine NTCDI) and Table S2 (for M-NTCDI, where M = Li^+^, Na^+^), show good agreement with experimental data, although an overestimation of the band frequencies is observed. This shift originates primarily from the harmonic approximation and the known tendency of certain exchange-correlation functionals, such as B3LYP, to overestimate the vibrational frequencies. For the frequency range analyzed in this work, 2000–1000 cm^–1^, the root-mean-square error between computed and experimental values of the neutral NTCDI is 25 cm^–1^ (without scaling of the computed frequencies). This value is consistent with the typical error range reported for unscaled DFT harmonic frequencies (∼20–50 cm^–1^), supporting the adequacy of our computational model.^36,37^ We also tested the M06–2X functional for comparison but found that it produced larger deviations from experimental frequencies in this system. For the pristine NTCDI molecule (Figure 5A), the group vibrations of −C=O bonds are easily recognizable and assigned from the atomic displacement patterns, whereas the remaining bands arise from coupled skeletal modes involving delocalized aromatic −C=C– and – CH–CH– bonds. Thus, we identified a ring mode corresponding to the vibration of all aromatic −C=C– and −CH–CH– bonds (1619 cm^–1^) and a mode where the aromatic bonds are also combined with deformations of the ternary imide and −CH_2_– groups (1277 cm^–1^), corresponding to the experimental band at 1251 cm^–1^. We also identify modes of the ternary imide: axial stretching at 1360 cm^–1^ consistent with the experimental band at 1355 cm^–1^ and low-intensity deformations at 1400 cm^–1^ coupled with deformations of the −CH_2_– and −CH–CH– groups, which we associate with the experimental band around 1390 cm^–1^.

**Figure 5:**
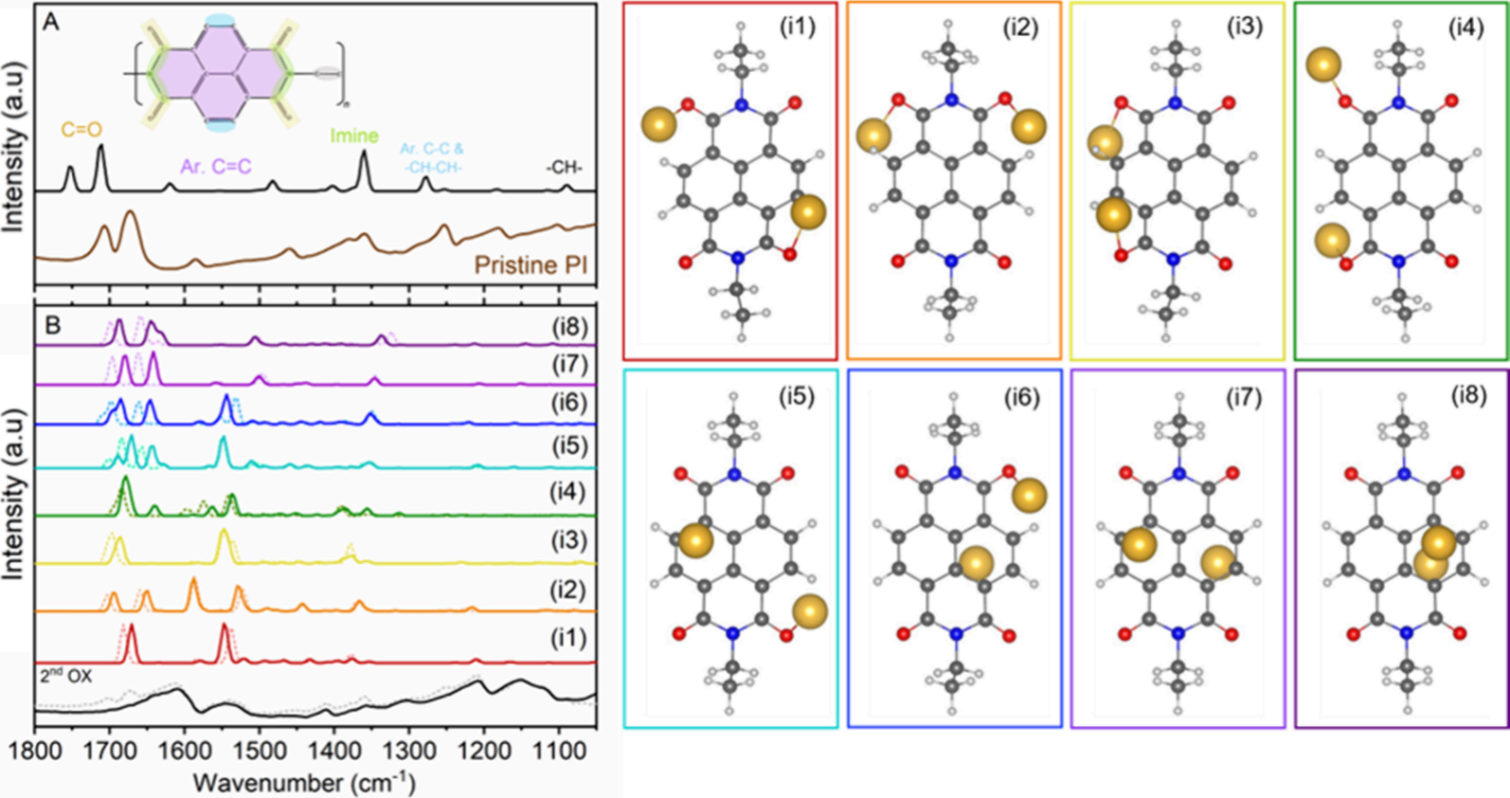
(A) SR-FTIR spectrum of a pristine PI electrode (brown) and NTCDI molecule (inset) showing assignment of main vibrational bands based on DFT calculated frequencies at the B3LYP/def2-TZVPP level (black). (B) SR-FTIR spectrum of Li/Na–PI at the end of second reduction (Na: blackline and Li: gray dashed line) and DFT calculated spectra for isomers i1-i8 of 2 Na-NTCDI (full lines) and 2 Li-NTCDI (dashed lines); structures of the corresponding isomers i1-i8 of the Na-NTCDI molecule. The color code for the atoms is blue for N, red for O, dark gray for C, light gray for H, and yellow for Na.

Figures 5B and S9 present comparisons between the experimental M-PI spectra, where M = Li^+^, Na^+^ (Li–PI corresponds to dashed lines), and the calculated spectra of 8 M-NTCDI isomers (relaxed structures are shown in Figure 5B for Na and Figure S9 for Li). The selected eight isomers for each metal ion illustrate representative structures contributing with different vibrational frequencies in the spectral region 1800–1050 cm^–1^ among all tested initial configurations. The 2 Na-NTCDI isomers, denoted i1–i8 in Figure 5B, and the 2 Li-NTCDI isomers in Figure S9B are ordered by increasing relative energy, with i1 being the most stable one for each chemistry (see Table S3). Structures i1–i8 can be grouped depending on the type of interaction between the Li/Na ions and the pristine molecule as i1–i4, where both metal ions are coordinated to a carbonyl group, i5 and i6, where one cation is coordinated to a carbonyl, while the other interacts with the aromatic ring, and i7 and i8, where both cations interacting mainly with the aromatic rings of the naphthalene moiety. The overall profiles of all the calculated and experimental spectra align well, though the frequency mismatch is slightly larger than for the pristine molecule. The most intense theoretical bands around 1700, 1540, and 1370 cm^–1^ differ among the eight 2 M-NTCDI isomers, reflecting symmetry variations induced by the M^+^ coordination geometry. For instance, the isomers i2 and i5–i8 show symmetric and asymmetric C=O vibrational modes (region 1705–1655 cm^–1^), while the spectra of isomers i1, i3, and i4 present only the asymmetric C=O vibrational mode. According to DFT calculations, these intense new bands in the reduced Li–PI and Na–PI correspond to vibrations of the unreacted carbonyl groups, significantly red-shifted by ∼50–60 cm^–1^ compared to the pristine state, such weakening being due to the Li^+^/Na^+^ coordination at adjacent carbonyl sites. Similar shifts have been reported for NTCDI molecules in the presence of metal ions (Li, Ni) by Han et al.^38^ The second most intense new band observed experimentally emerges at around 1543 cm^–1^. The very broad shape suggests multiple coupled modes might be contributing to its formation. It is reproduced in the calculated spectra as an intense peak at 1540 cm^–1^, corresponding to coupled vibration of aromatic −C–C–, −CH–CH– bonds, the imide and −CH_2_– deformations, and the −C–O^–^ stretching. Additional low-intensity bands appear at 1620–1580 cm^–1^, depending on the 2 Li-NTCDI isomer, assigned to ring vibrations only (aromatic −C–C– and −CH–CH– bonds). An illustration of the atomic displacements for the two principal bands in 2 Li-NTCDI isomers i1, i3, and i5 can be found in Figure S10.

Based on the calculated spectra, in addition to the enolization of the carbonyl groups in isomers i1–i4, the metal ion can also interact with the aromatic ring, as observed for isomers i5–i8. This interaction results in predicted vibrational modes at 1675, 1650, and 1550 cm^–1^ for spectra i5 and i6 in Figure 5B, which closely match the experimental bands in this region. The very broad shapes of the two main bands appearing in the experimental spectra upon PI reduction, centered between 1700 and 1500 cm^–1^, likely reflect overlapping contributions from multiple vibrational modes, consistent with a significant number of coexisting conformations within the polymeric structure. This suggests that several of the obtained monomeric isomers can represent local configurations within the polymer and contribute collectively to the observed spectral response. Based on the calculated relative energies and the Gibbs free energies of interaction (Table S3), the experimental bands could be result of the combination between monomeric units i1–i4, where the Li/Na ions are interacting exclusively with enol groups, but also combinations with isomers i5 and i6 cannot be discarded as their theoretical frequencies in the 1700–1500 cm^–1^ region show good agreement with the experimental bands. On the other hand, isomers i7 and i8, in which both cations interact exclusively with aromatic rings, are significantly less stable and fail to reproduce the intense band at 1550 cm^–1^ associated with coordinated carbonyls. Therefore, these monomeric units are considered to be unlikely to contribute to the experimental spectra. Mechanisms where Li interacts directly with aromatic groups have been reported in other studies on highly conjugated aromatic electrode materials.^39–42^

A careful examination of the major emerging band centered at 1630 cm^–1^ reveals differences between Li and Na cells (see Figure 4 and 3D plots in Figure S12). This broad band exhibits two maxima at 1605 and 1640 cm^–1^, which are particularly evident in Na cells. In Na cells, the 1640 cm^–1^ band appears at the early stages of reduction, while the 1605 cm^–1^ band becomes dominant at the final stages of reduction. For Li cells, the 1605 cm^–1^ band dominates at intermediate states of charge, but both bands reach their maximum intensity simultaneously at the end of charge (see Figures S13 and S14). The stepwise evolution in Na cells reverses upon oxidation and correlates with the two peaks observed by cyclic voltammetry (CV) and the two plateaus in the GCPL curve at −0.8 and −1.4 V vs. AC (Figure S1a vs Figure S1b), or especially during the second oxidation cycle (Figure 4b). The correlation between the electrochemical features with two peaks in CV and two plateaus (GCPL), and the two distinct IR bands suggests a sequential, two-step reaction mechanism in Na cells.

To further explore possible intermediates which could explain differences in redox mechanism in Li and Na cells, we also calculated the FTIR spectra of monocoordinated (radical-anion) species,^43–45^ formed upon one-electron reduction (Figure S11). Several stable isomers were identified, with the metal cation located near either a carbonyl or an aromatic region. Their calculated spectra resemble those of the bicoordinated complexes i1–i8, showing only slightly smaller red shifts. This spectral similarity may explain why the experimental bands are broad and overlapping, as both mono- and bicoordinated species may coexist, especially in the Na system, where weaker binding cooperativity could favor a higher population of monocoordinated intermediates before full reduction. Indeed, when examining the thermodynamics of successive cation binding (Table S4), both Li^+^ and Na^+^ coordination are exothermic processes, but the second coordination step (ΔΔ*H*_b,2_) is markedly more favorable for Li^+^ (−217 kJ/mol) than for Na^+^ (−142 kJ/mol). This difference reveals a higher degree of binding cooperativity in the Li system: the presence of one Li^+^ enhances the affinity for another, likely through local polarization of the carbonyl environment. For Na^+^, the weaker incremental stabilization suggests that each coordination event occurs more independently. The optimized geometries of Li- and Na-complexes are otherwise similar, indicating that this distinction arises from energetic rather than structural factors. Taken together, the energetic and spectroscopic data indicate that Li^+^ coordination is more cooperative and nearly concerted, driving the simultaneous reduction of adjacent carbonyls, whereas Na^+^ insertion proceeds in a more sequential manner through distinct intermediate states. It is worth noting that because the present calculations were carried out in the gas phase, the absolute stabilization energies should be interpreted with caution. Inclusion of solvation effects, for example via a CPCM continuum model with ε ≈ 1.4 (corresponding to diglyme solvent), would likely moderate the interaction energies and partially screen the cation–carbonyl interactions, but the relative trend (i.e., stronger Li^+^ binding and more progressive Na^+^ coordination) would remain unchanged.

Overall, operando FTIR and DFT analyses converge on a consistent mechanistic picture: Li^+^ drives a concerted, near-simultaneous coordination and reduction of neighboring carbonyls, while Na^+^ follows a stepwise two-stage pathway, reflected in the two distinct IR bands and electrochemical plateaus. Such differentiation between concerted and sequential redox behavior within the same polymeric framework has not been explicitly demonstrated for polyimide electrodes. The present analysis thus provides the first direct evidence linking the observed two-step spectral and electrochemical response to distinct coordination energetics of Li^+^ and Na^+^ within redox-active polyimide electrodes. These insights into the reaction mechanism are uniquely enabled by synchrotron-based FTIR spectroscopy, which provides the high spectral resolution and sensitivity necessary to capture subtle vibrational changes that would be challenging to detect with conventional FTIR techniques.

In contrast, during lithium coordination, the two plateaus in the electrochemical curve are almost imperceptible, and the two corresponding local maxima, though still visible, respond simultaneously. However, subtle differences in reactivity—consistent with the order observed in Na cells—can be detected with careful analysis (see Figure S12). Despite differences in the evolution of these bands during Na and Li uptake and release, the overall shape and position of the spectral maximum at the final stage of reduction (−2.1 V) are similar for both systems (see Figure S8). In the case of Ca cells, a closer examination of the 1570–1650 cm^–1^ region after reduction suggests that the band at (1640 cm^–1^) may have shifted to slightly lower energy, or that the corresponding redox reaction may not occur or is limited in Ca cells, potentially explaining the lower capacity observed in these cells (Figure S7).

### Fast Operando SR-FTIR

To leverage the fast acquisition rates allowed by synchrotron radiation, attempts were made to track the compositional changes associated with the electrochemical reaction at faster cycling rates. Operando SR-FTIR measurements of PI electrodes cycled in Na electrolyte at 1C, with an acquisition time of 60s (Figure 6), revealed capacities of 99 and 98 mAh/g upon reduction and oxidation, respectively. These results show notable differences in the evolution of the spectra compared to those of the previous cycles conducted on the same electrode at a C/4 rate, as depicted in Figure 4B. Upon reduction, a significant decrease in intensity was observed for the bands at 1703 and 1673 cm^–1^ (carbonyl bands) as well as at 1360 and 1255 cm^–1^, corresponding to the (C)_3_-N stretching and skeletal vibration. Concomitantly, a new band at 1525 cm^–1^ appeared. However, from the higher energy band, only the peak at 1640 cm^–1^ was detected. At a lower C rate, this band formed at the initial stages of reduction and was associated with the higher voltage plateau (Figure 4B, contour plot). As the C rate increased, the overall capacity decreased from 150 mAh/g to 100 mAh/g, and the band previously observed at slower rates, centered at 1605 cm^–1^, was no longer visible. These results highlight significant differences in the redox mechanisms depending on the cycling rate, correlating with the reversible capacity and demonstrating the potential of operando SR-FTIR to track electrochemical reactions at a technologically relevant C rate for battery applications (1C). From a technical perspective, this C rate is still well within the time resolution limit, considering that in these experimental conditions, with a 30 × 30 μm^2^ beam size, 0.2 s was sufficient to obtain a spectrum with good S/N, following reactions at rates of 20C would still allow us to measure 900 spectra per half cycle. However, faster rates may require specially designed electrodes that are thin and porous in order to minimize resistive and inhomogeneous current line distribution effects. Indeed, in the cell configuration used here, the sample is facing the counter electrode from the backside and reactivity gradient through the electrode thickness can occur. The test performed at 2.5C in Li cells resulted in unreadable data where several inversions of the bands could be observed throughout the cycles with little changes in the relevant IR bands (Figure S17).

**Figure 6:**
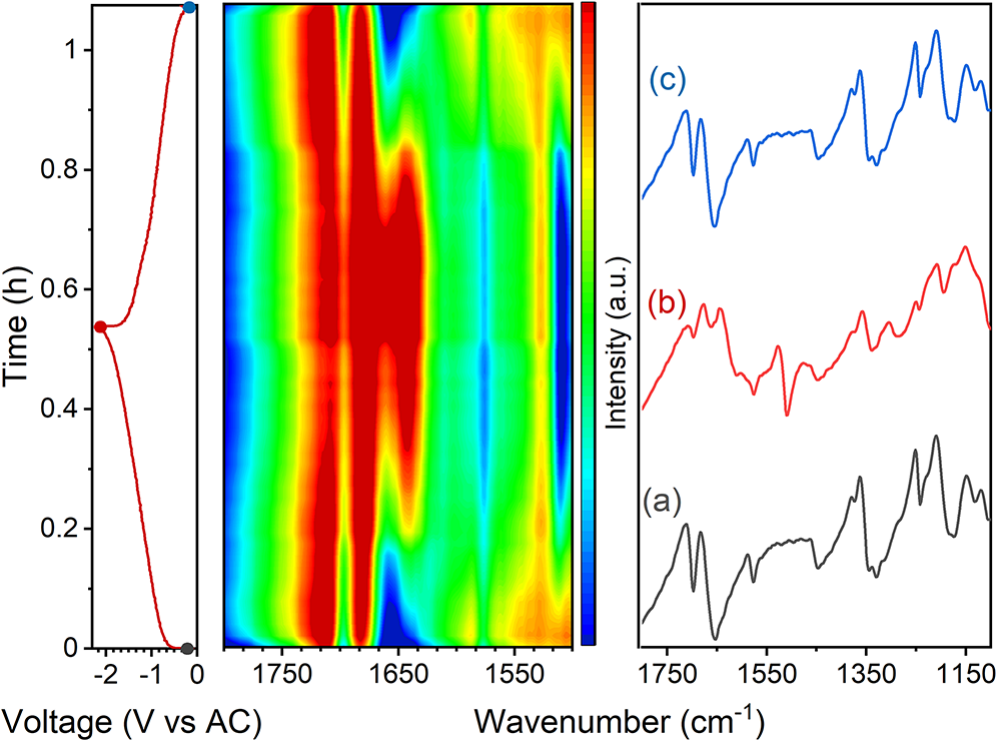
GCPL profile of PI at 1C in NaTFSI in DG, its corresponding contour plot of the operando SR-FTIR spectra in the region 1800–1500 cm^–1^ and the selected spectra corresponding to the initial state (a), end of reduction (−2.1 V vs. AC) (b), and end of oxidation (−0.1 V vs. AC) (c).

### Fast Operando SR-FTIR Microspectroscopy Mapping

To explore the capabilities of operando SR-FTIR, which allows for subsecond spectral acquisition with a spatial resolution of 10 μm, we conducted operando microspectroscopy mapping of the electrodes. Initially, PI electrodes were cycled in Li cells at a C/4 rate, and a large section of the electrode (190 × 250 μm^2^) was mapped with an IR beam spot size of 20 × 20 μm^2^, resulting in the acquisition of 154 spectra per map. With an acquisition time of 1 s per spectrum (5 scans), we obtained one map every 2 min, resulting in 194 maps over the full charge and discharge process (7 h at C/4).

Figure 7 shows a representative selection of 24 maps acquired during the operando charge and discharge of PI electrodes cycled in a Li cell. The color maps represent the integrated intensity in the region of 1508–1660 cm^–1^, corresponding to the two new bands formed upon reduction and simultaneous ion coordination. An example of the corresponding operando FTIR spectra for a single position is available in the Supporting Information (Figure S18), along with the operando microspectroscopy map of the bands corresponding to the carbonyl groups in the region of 1662–1750 cm^–1^ (Figure S19) and their single position spectra (Figures S20).

**Figure 7:**
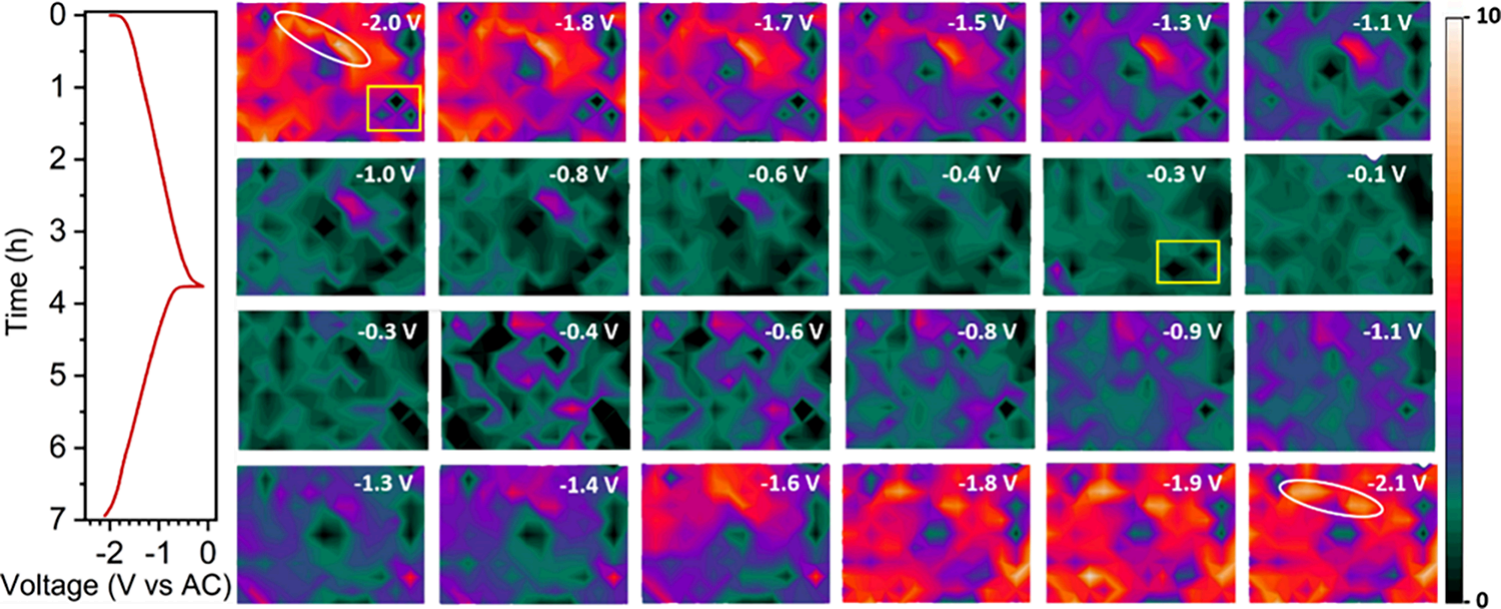
GCPL profile of PI at C/4 in LiTFSI in DG and a representative selection of the corresponding operando SR-FTIR microspectroscopy maps of the electrode at different stages of charge. Color scale corresponds to the chemical distribution of the IR bands appearing in the region (1508–1660 cm^–1^) in the scanned area of the electrode (190 × 250 μm).

This experiment began from a reduced state at −2.1 V vs AC, where the bands corresponding to the pristine carbonyl groups were absent, and those corresponding to neighboring C=O of the enolate groups, C–Li^+^ vibrations, and ring breathing vibrations were strong. The maps show a progressive decrease of these bands, at the expense of the growth of the C=O bands, ultimately recovering a spectrum with all the features of the pristine material at the end of oxidation (*t* = 3.8 h, cutoff = −0.1 V vs AC, 159 mAh/g). The reverse process was observed upon reduction, showing good reversibility of the material and associated spectral features (*t* = 6.9 h, cutoff = −2.1 V vs AC, 135 mAh/g). As seen in the single-point operando measurement (Figure 4A), a strong reversible change in the intensity of the bands at 1360 and 1255 cm^–1^, corresponding to (C)_3_–N stretching and skeletal vibration, was observed upon electrochemical cycling. These results reveal a high degree of reversibility of the PI electrode material and a fully reduced state (−2.1 V vs. AC) where the pristine carbonyl bands completely disappear.

The microspectroscopy mapping revealed an inhomogeneous spatial distribution of chemical features, believed to be related to the distribution of components of the electrode that are in focus and the limited sampling depth of the technique (1–3 μm). The mapping further showed that, although regions with initially high or low signal intensity could undergo substantial changes during cycling, the same spatial regions tended to recover their original signal characteristics after one full cycle, with high-intensity regions returning to high-intensity and low-intensity regions returning to low intensity (white ellipses and yellow rectangles in Figure 7).

To explore the technique’s limits, operando SR-FTIR microspectroscopy mapping was performed at faster cycling rates (2.5C) on a Na cell, mapping a smaller region of an electrode (150 × 95 μm^2^) with a beam size of 20 × 20 μm^2^, leading to the acquisition of 42 spectra per map. With an acquisition time of 1 s per spectrum, one map was obtained every 53 s, resulting in 15 maps over the full charge and discharge process and 45 maps over three consecutive cycles in 40 min. Under these experimental conditions, the PI electrodes cycled in Na cells showed a significant capacity drop, with achieved capacities oscillating between 43 and 53 mAh/g (Figure 8), far below the 159 mAh/g obtained at C/4 (Figure 4B) and 99 mAh/g obtained at 1C (Figure 6).

**Figure 8:**
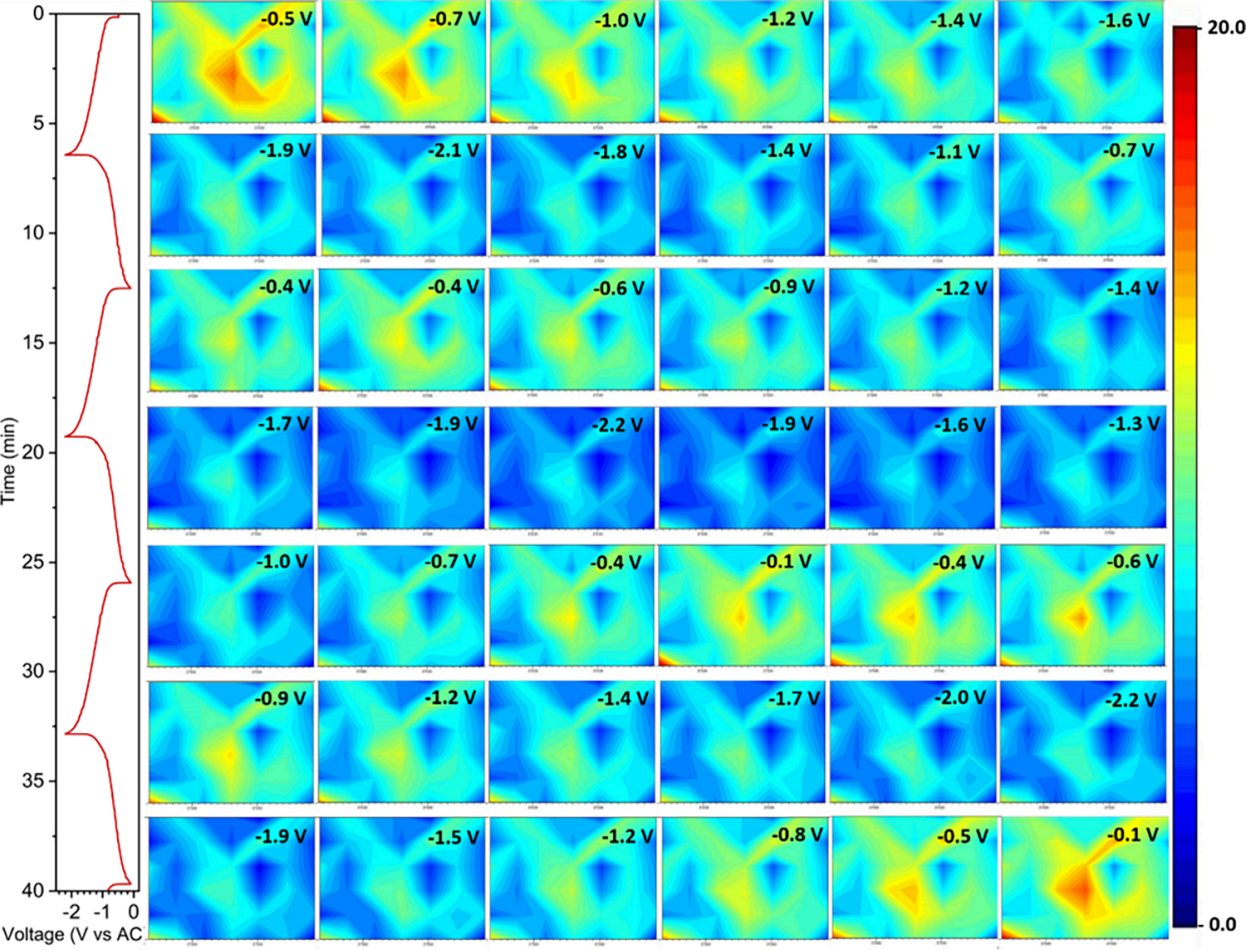
GCPL profile of PI at 2.5C in NaTFSI in DG and the corresponding *operando* SR-FTIR microspectroscopy maps of the electrode at different states of charge. Color scale corresponds to the chemical distribution of the IR bands appearing in the region (1660–1850 cm^–1^) in the scanned area of the electrode (150 × 95 μm^2^).

Consequently, the operando SR-FTIR microspectroscopy mapping showed proportionally lower changes in the integrated intensities of the bands corresponding to carbonyl groups (1660–1850 cm^–1^), as seen in Figure 8 and its corresponding example of a spectrum at a single position (Figure S22). For clarity, the operando mapping of the new band at lower energy (1510–1570 cm^–1^) is also depicted (Figure S22), along with its corresponding example spectra (Figure S23). Consistent with the results shown in Figure 6 (1C), the upper energy band assigned to the new C=O–N–C–O^–^–Na^+^ only shows the band at 1640 cm^–1^, as expected from the low achieved capacities. Compared to the single position operando measurement, mapping captured a region with a local maximum signal, allowing us to follow reactivity in a wider area of the electrode and ensure capturing changes in a region that maintains a good signal-to-noise ratio throughout the entire cycling, regardless of possible intensity changes due to swelling or expansion of the electrode.

Finally, it is worth mentioning that these results were obtained by measuring the back of the electrode, which is both farther away from the counter electrode and from the copper mesh current collector (Figure S2), thus experiencing the highest impedance and most limited mass transport. Nonetheless, in Figure 6 we can clearly observe evidence for the enolation process taking place even at 1C confirming that high-rate processes can be captured using the electrochemical setup presented in his work.

## Conclusions

The development of the synchrotron-based *operando* Fourier transform infrared (SR-μFTIR) microspectroscopy technique has enabled real-time chemical analysis and imaging at the micrometer scale of organic electrode materials during electrochemical reactions occurring in a battery. This technique provides unique insights into the redox reaction mechanism of noncrystalline, lightweight element-based materials, where other techniques, such as X-ray diffraction or X-ray absorption, are either inapplicable or require complex experimental setups.

*Operando* SR-μFTIR conducted on PI electrodes in Li, Na, and Ca cells has revealed a reaction mechanism characterized by the decrease of all major IR active bands of the pristine PI, alongside the concomitant appearance of two intense broad bands centered at 1630 and 1540 cm^–1^. Simulated spectra obtained by DFT calculations confirmed the assignment of the major IR active bands in pristine PI. Furthermore, the simulated spectra of two 2Li–PI isomers aligned well with the observed IR bands, depicting a complex reaction mechanism in which the enolation reaction induces a strong red shift of the carbonyl groups connected to the same nitrogen of the imide while also inducing a great change in the aromatic ring vibrations.

The high spectral quality of SR-μFTIR enabled the identification of key differences in the reaction mechanism between Li and Na cells. In Na cells, the broad band centered at 1630 cm^–1^ showed a fine structure that correlates with the state of charge and the pseudoplateau-like features observed in the GCPL profile. The presence of these multiple IR active bands in the region (1550 to 1650 cm^–1^) is consistent with the DFT model, suggesting that one of the coordinating ions is located above the aromatic ring. The strong stabilization of the aromatic ring by charge delocalization results in several intense symmetric and asymmetric (C=O) vibrational modes.

The superior spatial coherence and large photon flux yield of the synchrotron radiation allowed us to follow the electrochemical reactions at high C rate (2.5C), where the limitation arose from the rate capability of the active material rather than the *operando* FTIR technique. *Operando* experiments conducted at fast rates revealed less significant composition changes associated with the limited extent of the electrochemical process, which correlate with the observed capacity drop.

The *operando* FTIR microspectroscopy images, where each pixel represents a complete infrared spectrum, enabled tracking of variations in both space and time. In contrast, conventional IR sources exhibit weak intensity at high spatial resolution, requiring long acquisition times to achieve an acceptable signal-to-noise ratio, making raster scanning time-consuming. Synchrotron-based FTIR imaging, however, enables the rapid acquisition of hundreds of measurements over large electrode areas with improved spatial resolution, making it a promising and powerful alternative for detecting heterogeneities. Therefore, coupling synchrotron light to FTIR microspectroscopy to perform raster scanning images enables a better understanding of the electrode redox activity. Moreover, tracking the reactivity across a wider area of the electrode ensures capturing changes in a region that maintains a good signal-to-noise ratio upon cycling. This is particularly important for electrode materials prone to swelling, where significant intensity changes can occur. Relying solely on single-point measurements can lead to misleading interpretations of reaction dynamics. The superior spatial resolution provided by synchrotron radiation can also become crucial when investigating inhomogeneous layers, such as SEI or poorly formulated electrodes (e.g., poor distribution of carbon additive), presenting inhomogeneous reactivity.

While the present work focuses on a limited spectral window, the intrinsic potential of synchrotron radiation to access a much wider range opens exciting prospects for extending future operando studies into the far-infrared region, thereby deepening the understanding of electrode–electrolyte interactions. In future work, implementing variable-angle reflectance measurements at higher incidence angles could provide tunable probing depths, enabling the assessment of possible chemical or electrochemical gradients across the electrode thickness. Such an approach would complement the present near-surface analysis and offer a more comprehensive view of the reaction distribution within thick electrodes.

This powerful and noninvasive characterization method is conducted by using a commercially available electrochemical cell, which closely mimics real operating conditions with minimal interference from the analytical technique. As a result, the reliability and relevance of the obtained analytical data are significantly enhanced. We envision that synchrotron-based operando FTIR spectroscopy is expected to find broad applicability in the battery research community, offering detailed and meaningful insights into the dynamic processes within batteries and aiding the development of next-generation energy storage systems.

## Supplementary Material



## References

[1] Aurbach D., Zaban A., Schechter A., Ein-Eli Y., Zinigrad E., Markovsky B. (1995). The Study of Electrolyte Solutions Based on Ethylene
and Diethyl Carbonates for Rechargeable Li Batteries: II. Graphite
Electrodes. J. Electrochem. Soc..

[2] Aurbach D., Pollak E., Elazari R., Salitra G., Kelley C. S., Affinito J. (2009). On the Surface Chemical Aspects of Very High Energy
Density, Rechargeable Li–Sulfur Batteries. J. Electrochem. Soc..

[3] Zhuang G. V., Xu K., Yang H., Jow T. R., Ross P. N. (2005). Lithium Ethylene
Dicarbonate Identified as the Primary Product of Chemical and Electrochemical
Reduction of EC in 1.2 M LiPF 6/EC:EMC Electrolyte. J. Phys. Chem. B.

[4] Vizintin A., Bitenc J., Kopač Lautar A., Pirnat K., Grdadolnik J., Stare J., Randon-Vitanova A., Dominko R. (2018). Probing Electrochemical
Reactions in Organic Cathode Materials via in Operando Infrared Spectroscopy. Nat. Commun. 2018 91.

[5] Xu, K. Electrolytes and Interphases in Li-Ion Batteries and Beyond. Chem. Rev.. American Chemical Society December 10, 2014; pp 114 11503–11618. 10.1021/cr500003w. 25351820

[6] Seo D. M., Reininger S., Kutcher M., Redmond K., Euler W. B., Lucht B. L. (2015). Role of Mixed Solvation and Ion Pairing in the Solution
Structure of Lithium Ion Battery Electrolytes. J. Phys. Chem. C.

[7] Black A. P., Sorrentino A., Fauth F., Yousef I., Simonelli L., Frontera C., Ponrouch A., Tonti D., Palacín M. R. (2023). Synchrotron
Radiation Based Operando Characterization of Battery Materials. Chem. Sci..

[8] Goren E., Chusid Youngman O., Aurbach D. (1991). The Application of
In Situ FTIR Spectroscopy to the Study of Surface Films Formed on
Lithium and Noble Metals at Low Potentials in Li Battery Electrolytes. J. Electrochem. Soc..

[9] Santos É. A., Anchieta C. G., Fernandes R. C., Pinzón C M. J., Miranda A. N., Galantini I., Maia F. C. B., Doubek G., Rodella C. B., Da Silva L. M., Zanin H. (2023). Combining in Situ Electrochemistry,
Operando FTIR and Post-Mortem Analyses to Understand Co-Mn-Al Spinels
on Mitigating Shuttle Effect in Lithium-Sulfur Battery. Nano Energy.

[10] Akita Y., Segawa M., Munakata H., Kanamura K. (2013). In-Situ Fourier
Transform
Infrared Spectroscopic Analysis on Dynamic Behavior of Electrolyte
Solution on LiFePO4 Cathode. J. Power Sources.

[11] Matsushita T., Dokko K., Kanamura K. (2005). In Situ FT-IR
Measurement for Electrochemical
Oxidation of Electrolyte with Ethylene Carbonate and Diethyl Carbonate
on Cathode Active Material Used in Rechargeable Lithium Batteries. J. Power Sources.

[12] Möller K. C., Santner H. J., Kern W., Yamaguchi S., Besenhard J. O., Winter M. (2003). In Situ Characterization
of the SEI
Formation on Graphite in the Presence of a Vinylene Group Containing
Film-Forming Electrolyte Additives. J. Power
Sources.

[13] Matsui M., Dokko K., Kanamura K. (2008). Dynamic Behavior of
Surface Film
on LiCoO2 Thin Film Electrode. J. Power Sources.

[14] Shi F., Ross P. N., Zhao H., Liu G., Somorjai G. A., Komvopoulos K. (2015). A Catalytic
Path for Electrolyte Reduction in Lithium-Ion
Cells Revealed by in Situ Attenuated Total Reflection-Fourier Transform
Infrared Spectroscopy. J. Am. Chem. Soc..

[15] Vivek J. P., Berry N., Papageorgiou G., Nichols R. J., Hardwick L. J. (2016). Mechanistic
Insight into the Superoxide Induced Ring Opening in Propylene Carbonate
Based Electrolytes Using in Situ Surface-Enhanced Infrared Spectroscopy. J. Am. Chem. Soc..

[16] Wu L., Hu J., Chen S., Yang X., Liu L., Foord J. S., Pobedinskas P., Haenen K., Hou H., Yang J. (2023). Lithium Nitrate
Mediated Dynamic Formation of Solid Electrolyte Interphase Revealed
by in Situ Fourier Transform Infrared Spectroscopy. Electrochim. Acta.

[17] Hongyou K., Hattori T., Nagai Y., Tanaka T., Nii H., Shoda K. (2013). Dynamic in Situ Fourier
Transform Infrared Measurements of Chemical
Bonds of Electrolyte Solvents during the Initial Charging Process
in a Li Ion Battery. J. Power Sources.

[18] Kanamura K., Toriyama S., Shiraishi S., Ohashi M., Takehara Z. I. (1996). Studies
on Electrochemical Oxidation of Non-Aqueous Electrolyte on the LiCoO2
Thin Film Electrode. J. Electroanal. Chem..

[19] Bitenc J., Vizintin A., Grdadolnik J., Dominko R. (2019). Tracking Electrochemical
Reactions inside Organic Electrodes by Operando IR Spectroscopy. Energy Storage Mater..

[20] Imhof R., Novák P. (1998). In Situ Investigation of the Electrochemical
Reduction
of Carbonate Electrolyte Solutions at Graphite Electrodes To. J. Electrochem. Soc..

[21] Zhang Y., Katayama Y., Tatara R., Giordano L., Yu Y., Fraggedakis D., Sun J. G., Maglia F., Jung R., Bazant M. Z., Shao-Horn Y. (2020). Revealing Electrolyte Oxidation via
Carbonate Dehydrogenation on Ni-Based Oxides in Li-Ion Batteries by
in Situ Fourier Transform Infrared Spectroscopy. Energy Environ. Sci..

[22] Bechtel H. A., Johnson S. C., Khatib O., Muller E. A., Raschke M. B. (2020). Synchrotron
Infrared Nano-Spectroscopy and -Imaging. Surf.
Sci. Rep..

[23] Forero-Saboya J., Davoisne C., Dedryvère R., Yousef I., Canepa P., Ponrouch A. (2020). Understanding the Nature
of the Passivation Layer Enabling
Reversible Calcium Plating. Energy Environ.
Sci..

[24] Patil N., Medina-Santos J. I., García-Quismondo E., Goujon N., Mecerreyes D., Palma J., Marcilla R. (2025). Advancing Polyimide
Electrodes from Half-Cells to Pouch Cells: Balancing $/KWh, Stability,
and Scalability for Practical Li-Ion Organic Batteries. Energy Storage Mater..

[25] Nishide H. (2022). Organic Redox
Polymers as Electrochemical Energy Materials. Green Chem..

[26] Patil N., Mavrandonakis A., Jérôme C., Detrembleur C., Palma J., Marcilla R. (2019). Polymers Bearing Catechol Pendants
as Universal Hosts for Aqueous Rechargeable H+, Li-Ion, and Post-Li-Ion
(Mono-, Di-, and Trivalent) Batteries. ACS Appl.
Energy Mater..

[27] Zhu L., Ding G., Xie L., Cao X., Liu J., Lei X., Ma J. (2019). Conjugated
Carbonyl Compounds as High-Performance Cathode
Materials for Rechargeable Batteries. Chem.
Mater..

[28] Gannett C. N., Kim J., Tirtariyadi D., Milner P. J., Abruña H. D. (2022). Investigation
of Ion-Electrode Interactions of Linear Polyimides and Alkali Metal
Ions for next Generation Alternative-Ion Batteries. Chem. Sci..

[29] Hernández G., Casado N., Coste R., Shanmukaraj D., Rubatat L., Armand M., Mecerreyes D. (2015). Redox-Active
Polyimide-Polyether Block Copolymers as Electrode Materials for Lithium
Batteries. RSC Adv..

[30] Monti D., Patil N., Black A. P., Raptis D., Mavrandonakis A., Froudakis G. E., Yousef I., Goujon N., Mecerreyes D., Marcilla R., Ponrouch A. (2023). Polyimides as Promising Cathodes
for Metal-Organic Batteries: A Comparison between Divalent (Ca2+,
Mg2+) and Monovalent (Li+, Na+) Cations. ACS
Appl. Energy Mater..

[31] Verrelli R., Black A., Dugas R., Tchitchekova D., Ponrouch A., Palacin M. R. (2020). Steps Towards the Use of TiS2 Electrodes
in Ca Batteries. J. Electrochem. Soc..

[32] Hanwell M. D., Curtis D. E., Lonie D. C., Vandermeersch T., Zurek E., Hutchison G. R. (2012). Avogadro: An Advanced Semantic Chemical
Editor, Visualization, and Analysis Platform. J. Cheminform..

[33] Neese F. (2012). The ORCA Program
System. Wiley Interdiscip. Rev. Comput. Mol.
Sci..

[34] Weigend F., Ahlrichs R. (2005). Balanced Basis Sets of Split Valence, Triple Zeta Valence
and Quadruple Zeta Valence Quality for H to Rn: Design and Assessment
of Accuracy. Phys. Chem. Chem. Phys..

[35] Filmetrics. Spectral Thin Film Reflectance Calculator for Thin-Film Stacks. https://www.filmetrics.com/reflectance-calculator (accessed 2025–03–19).

[36] Merrick J. P., Moran D., Radom L. (2007). An Evaluation of Harmonic Vibrational
Frequency Scale Factors. J. Phys. Chem. A.

[37] Wong M. W. (1996). Vibrational
Frequency Prediction Using Density Functional Theory. Chem. Phys. Lett..

[38] Han X., Yi F., Sun T., Sun J. (2012). Synthesis and Electrochemical Performance
of Li and Ni 1,4,5,8-Naphthalenetetracarboxylates as Anodes for Li-Ion
Batteries. Electrochem. commun..

[39] Han X., Qing G., Sun J., Sun T. (2012). How Many Lithium Ions
Can Be Inserted onto Fused C6 Aromatic Ring Systems?. Angew. Chemie Int. Ed..

[40] Lubis A. L., Baskoro F., Lin T. H., Wong H. Q., Liou G. S., Yen H. J. (2023). Redox-Active High-Performance
Polyimides as Versatile
Electrode Materials for Organic Lithium- and Sodium-Ion Batteries. ACS Appl. Mater. Interfaces.

[41] Baskoro F., Lin H. J., Chang C. W., Wang C. L., Lubis A. L., Yen H. J. (2023). High-Performance
Aramid Electrodes for High-Rate and
Long Cycle-Life Organic Li-Ion Batteries. J.
Mater. Chem. A.

[42] Tao L., Zhao J., Chen J., Ou C., Lv W., Zhong S. (2021). 1,4,5,8-Naphthalenetetracarboxylic Dianhydride Grafted Phthalocyanine
Macromolecules as an Anode Material for Lithium Ion Batteries. Nanoscale Adv..

[43] Gu S., Chen J., Hao R., Chen X., Wang Z., Hussain I., Liu G., Liu K., Gan Q., Li Z., Guo H., Li Y., Huang H., Liao K., Zhang K., Lu Z. (2023). Redox of Anionic
and Cationic Radical
Intermediates in a Bipolar Polyimide COF for High-Performance Dual-Ion
Organic Batteries. Chem. Eng. J..

[44] Wang J., Liu H., Du C., Liu B., Guan H., Liu Y., Guan S., Sun Z., Yao H. (2024). Towards High Performance
Polyimide Cathode Materials for Lithium-Organic Batteries by Regulating
Active-Site Density, Accessibility, and Reactivity. eScience.

[45] Bai Y., Wang Z., Qin N., Ma D., Fu W., Lu Z., Pan X. (2023). Two-Step Redox in Polyimide:
Witness by In Situ Electron
Paramagnetic Resonance in Lithium-Ion Batteries. Angew. Chem., Int. Ed..

