# Supplementary material for: Operando Synchrotron-Based Fourier Transform Infrared Microspectroscopy of Metal-Ion Organic Battery Materials
Source: Chem Mater. 2026 Jan 7;38(2):645–56. doi: 10.1021/acs.chemmater.5c01795 (PMC12854678; doi:10.1021/acs.chemmater.5c01795)
Supplement: Supplementary file 1 [file cm5c01795_si_001.pdf]

Operando Synchrotron-based Fourier Transform Infrared Microspectroscopy of Metal-ion Organic Battery Materials

Ashley P. Black,[a†] Deyana S. Tchitchekova,[a†] Nagaraj Patil,[c] Nicolas Goujon,[d,f,g] David Mecerreyes,[d] Rebeca Marcilla,[c] Ibraheem Yousef,[b*] Alexandre Ponrouch[a,b*]

[a] *Institut de Ciència de Materials de Barcelona, ICMAB-CSIC, Campus UAB, 08193 Bellaterra, Catalonia, Spain.*

[b] *ALISTORE – European Research Institute –CNRSFR 3104, 80039, Amiens, France.*

[c] *CELLS - ALBA synchrotron, 08290 Cerdanyola del Vallès, Catalonia, Spain.*

[d] *Electrochemical Processes Unit, IMDEA Energy, Avda. Ramon de la Sagra 3, 28935, Mostoles, Spain.*

[e] *POLYMAT University of the Basque Country, Avda. Tolosa 72, 20018 Donostia-San Sebastian, Spain.*

[f] *Applied Chemistry Department, Faculty of Chemistry, University of the Basque Country EHU, 20018 San Sebastián, Spain.*

[g] *Ikerbasque, Basque Foundation for Science, 48013 Bilbao, Spain.*

† *Equal contribution*

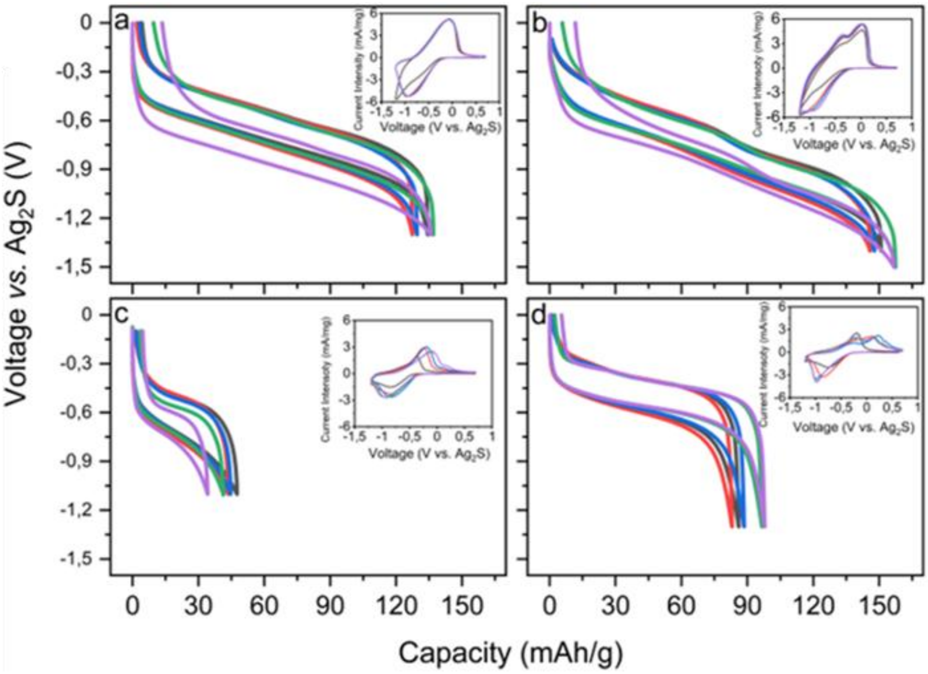

Figure. S1 GCPL potential (vs Ag/Ag$_2$S) vs capacity curves of PI in 1 M (a) LiTFSI and (b) NaTFSI and 0.5 M (c) Mg(TFSI)$_2$ and (d) Ca(TFSI)$_2$ in EC:PC for the last cycles at 1C, C/2, C/10, and C/20 rates and CVs (cycles 1, 5, 10, 20, and 30 at 5 mV/s) obtained using the same electrolytes. Adapted from [1] with permission from the American Chemical Society.

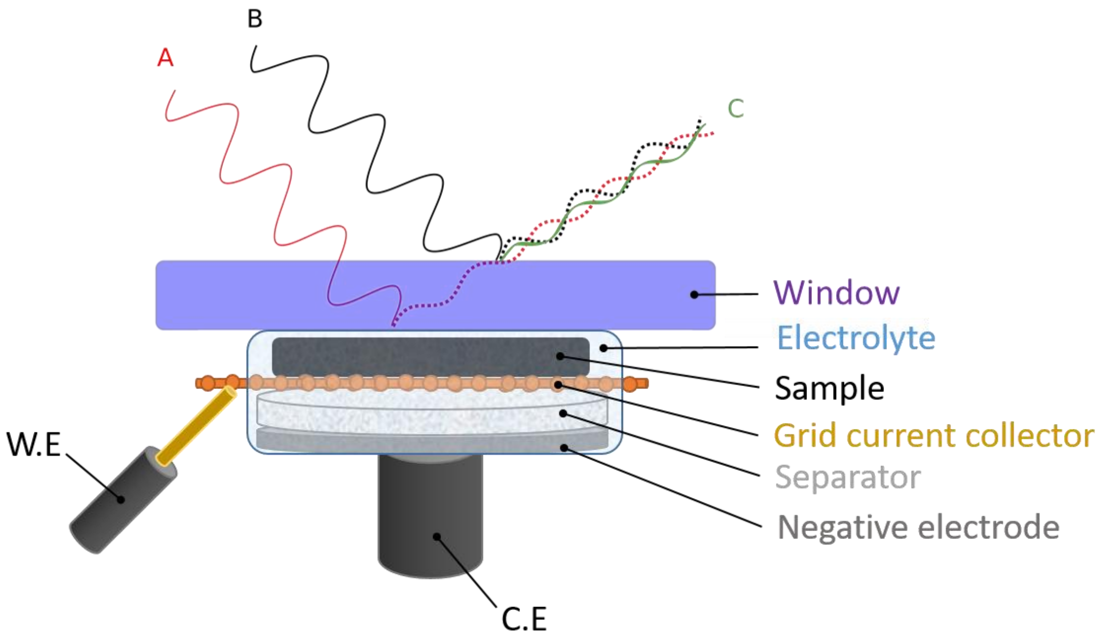

Figure. S2 Schematic of the electrochemical cell and partial reflection of the light by two surfaces of the window. Depending on the wavelength of the radiation, refractive index of the material, the window thickness and the angle of the penetrating radiation this interaction can result in constructive or destructive interference. A and B are two incoming radiation and C is the resulting interference radiation.

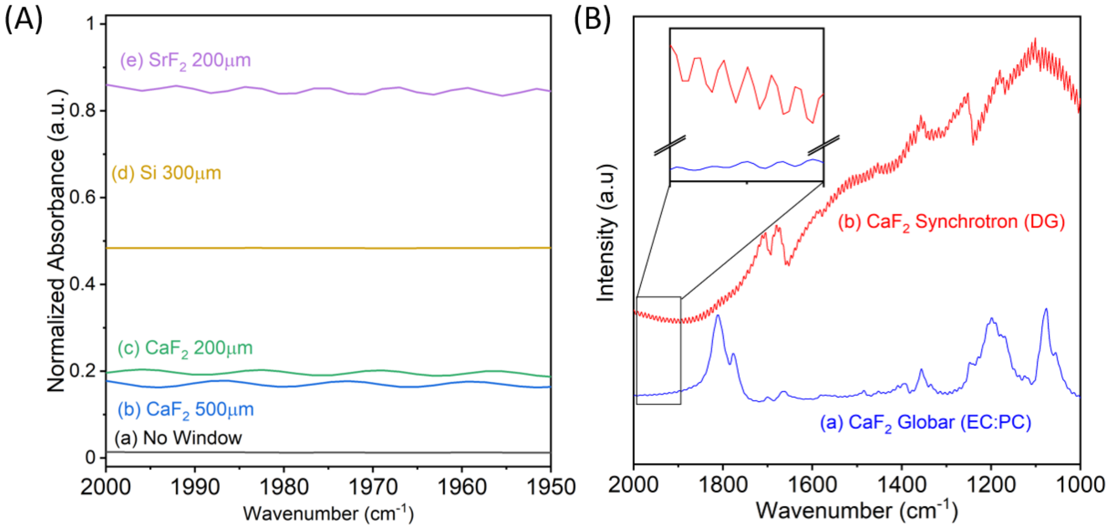

Figure. S3 (A) zoom in on the IR spectra of PI in the region 2000-1950 showing the frequency of the interference pattern with no window (a) and with a 500µm CaF_2 (b), 200µm CaF_2 (c), 300µm Si (d) or a 200µm SrF_2 (e) window. (B) IR spectra of PI electrodes using a CaF_2 window measured with a Globar source (a) and with the synchrotron source (b). band at 1800 in (a) are from carbonyl group of the EC:PC solvent of the electrolyte.

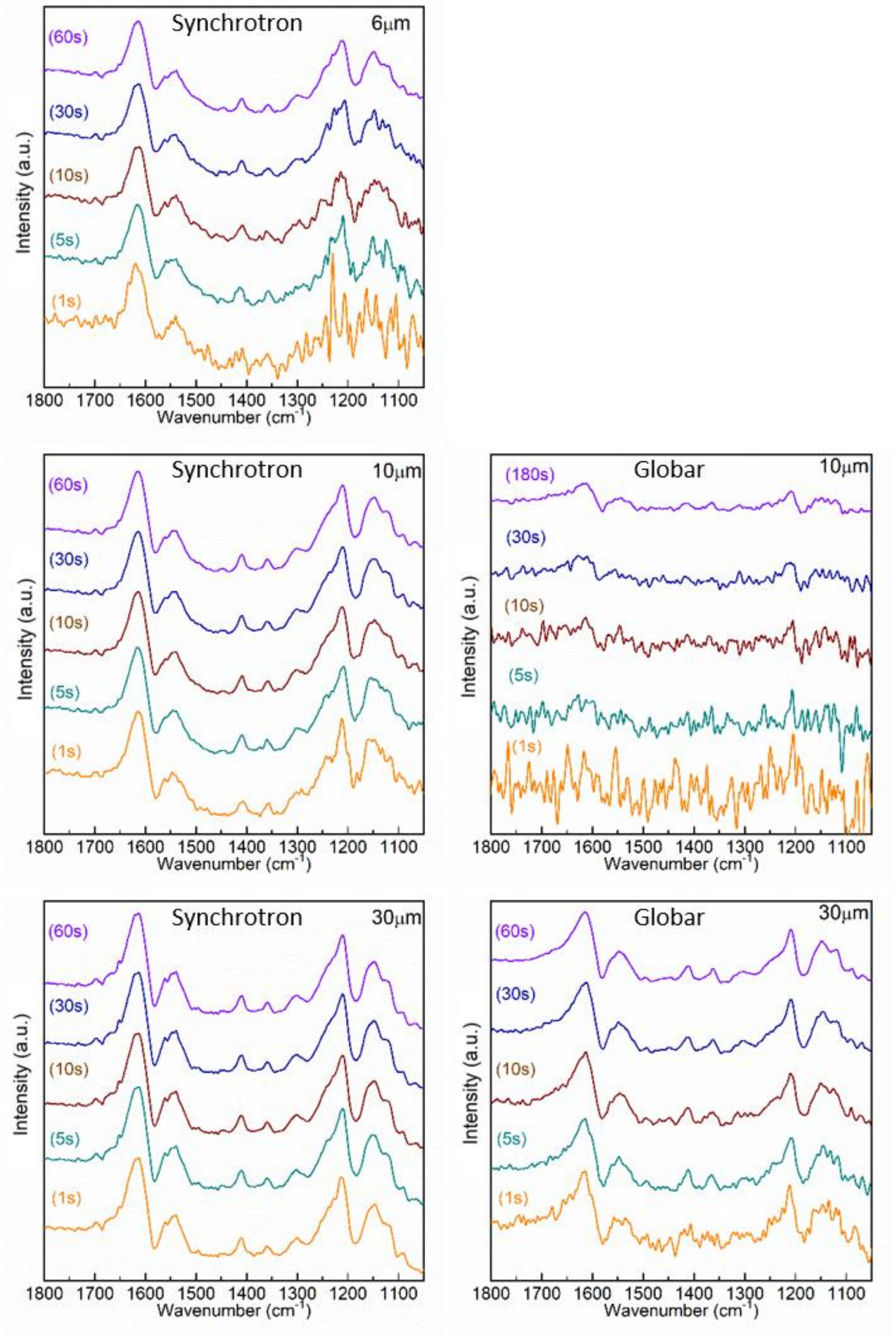

Figure. S4 FTIR spectrums measured with IR light spot sizes of 30x30 μm^2, 10x10 μm^2 and 6x6 μm^2 and accumulating 1, 5, 10, 30, 60, 180 scans with Globar and Synchrotron source.

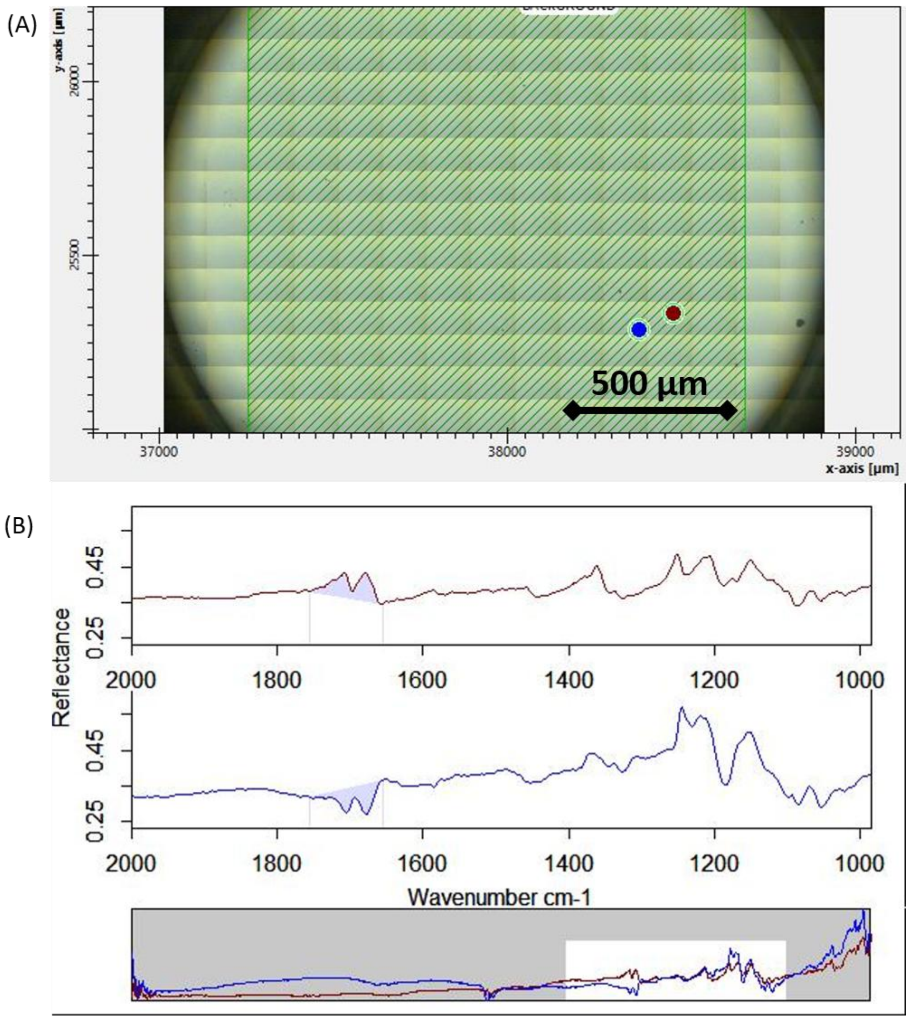

Figure. S5 (A) Image of the mapped area, brown and blue dots show the location of two areas where the light interacts in absorption (brown) and reflection (blue) with the sample. (B) the corresponding IR spectra.

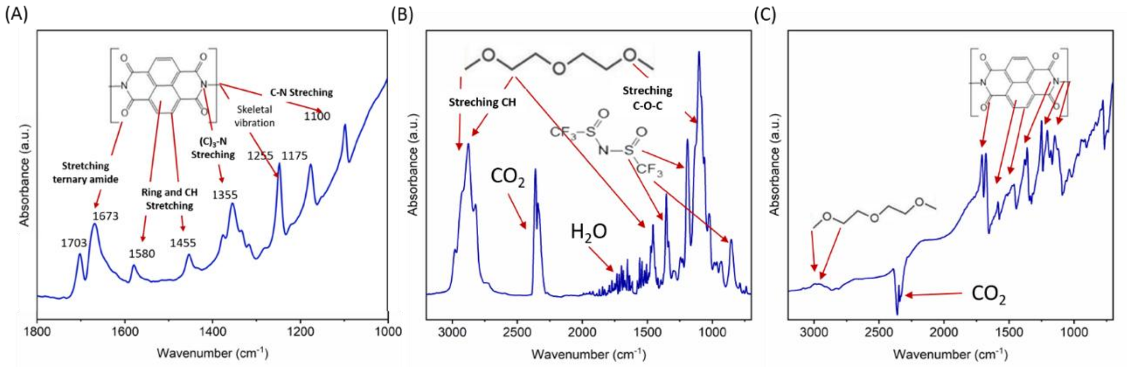

Figure. S6 SR-FTIR spectra's of dry PTCDA electrode (A), Electrolyte solution 1M NaTFSI in DG (B), ElCell containing PTCDA electrode and 100 µm of 1M NaTFSI in DG electrolyte (C) and their corresponding assigned vibrational bands. *(No relevant bands from SWCNT+RGO are visible in spectra (A) and signals from the electrolyte in (C) are very week if any).*

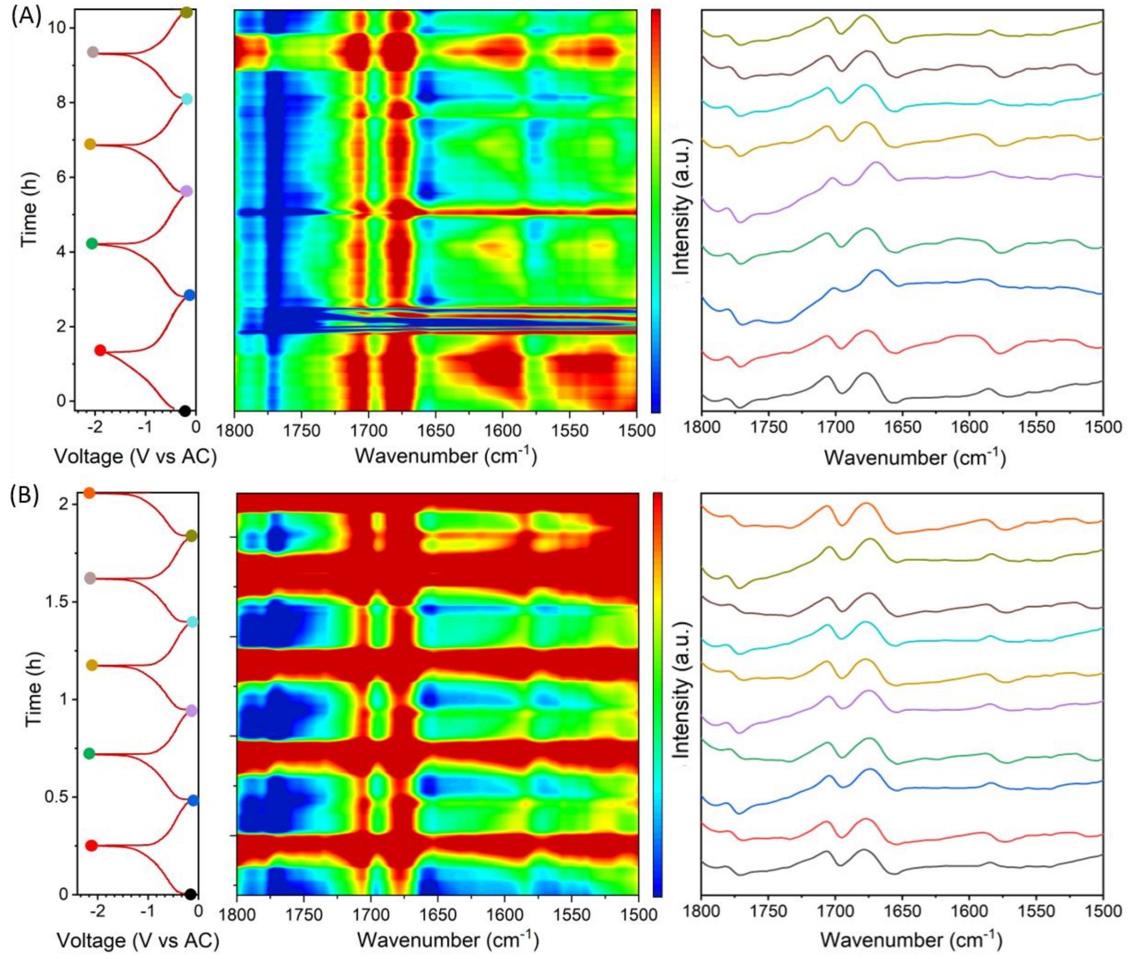

Figure. S7 The GCPL profile of PI in $Ca(TFSI)_2$ in DG at C/4 (A) and at 1C (B), their corresponding contour plot of the operando SR-FTIR spectra in the region 1500-1800 cm^{-1} and the selected spectra corresponding to the initial state, end of reduction and second oxidation of several consecutive electrochemical cycles.

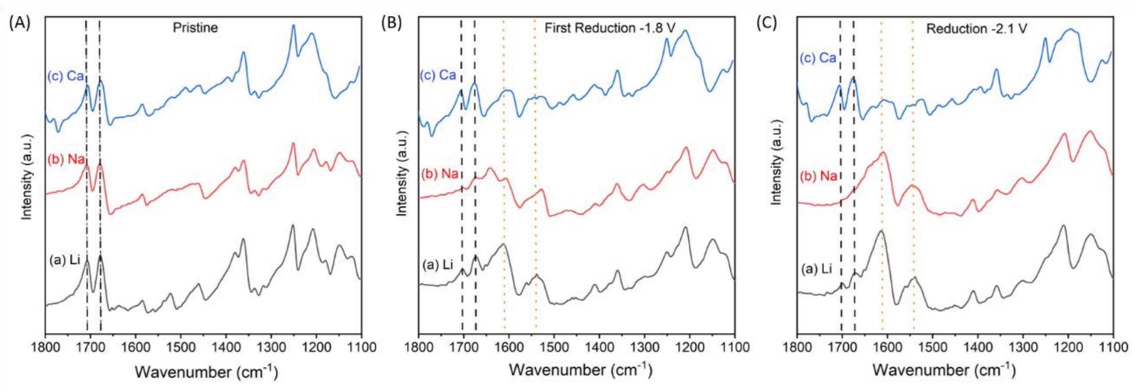

Figure. S8 Comparative SR-FTIR spectra of PI in Li, Na and Ca cells at pristine stage (A) and at the end of reduction to -1.8 V vs. AC (B) and at lower reduction cutoff of -2.1 V vs. AC (C).

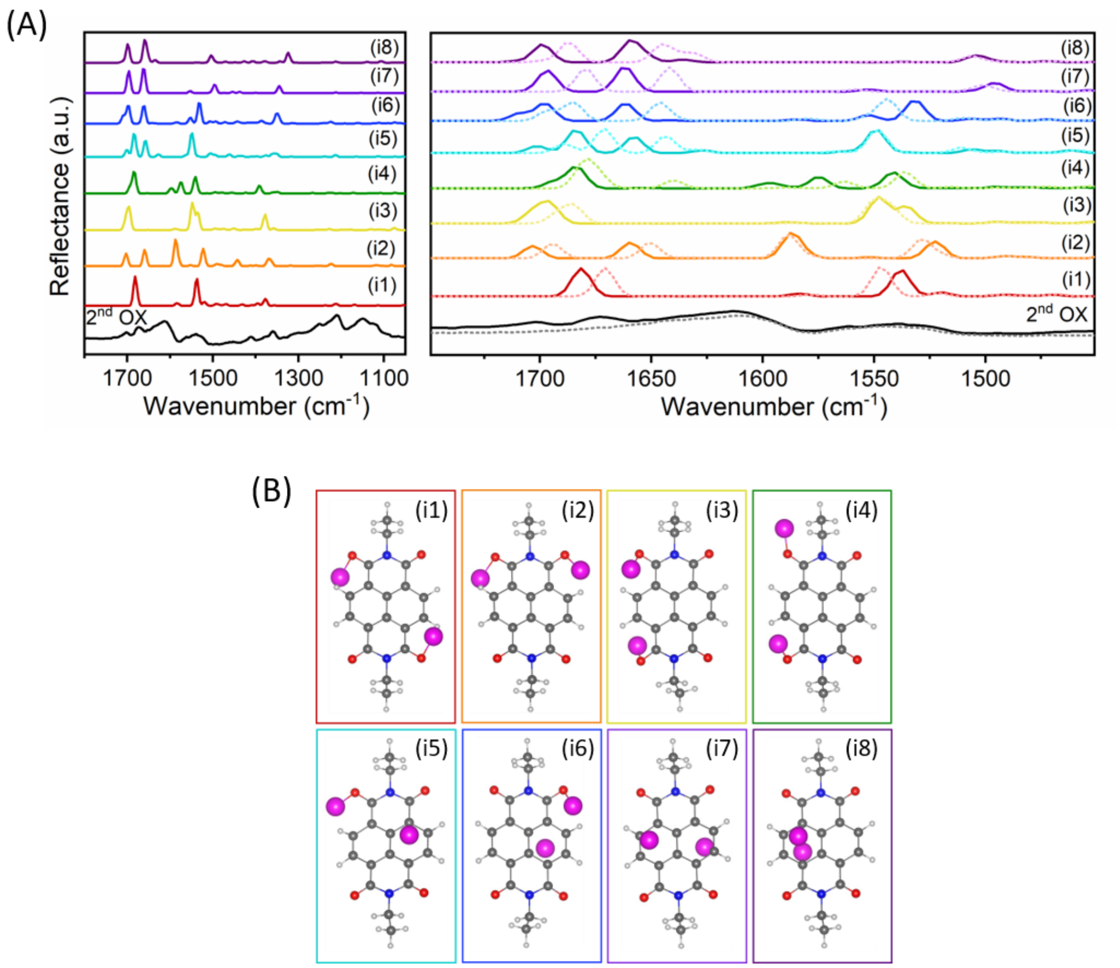

Figure. S9 (A) SR-FTIR spectrum of Li-PI at the end of second reduction (black) and DFT calculated spectra for isomers (i1-i8) of 2xLi-NTCDI. The amplified region 1750-1450 cm^{-1} includes the calculated 2xNa-NTCDI spectra as well (dashed lines) for easier comparison. The corresponding 2xLi-NTCDI isomers are displayed in (B). The color code for the atoms is: blue for N, red for O, dark grey for C, light grey for H, and pink for Li.

Table S1. Assignment of experimental and DFT calculated characteristic IR bands of pristine PI and NTCDI, respectively. Matching of the vibrational modes was achieved by visual inspection of the atomic displacements in internal coordinates.

Group	PI	NTCDI
	Exp.	DFT
ν(C=O) sym.	1706	1752
ν(C=O) asym.	1675	1711
Ring	1583	1619
δ(CH_2)	1455	1482
δ(CN, CHCH, CH_2)	1380	1400
ν(CN), δ(CHCH, CH_2)	1360	1360
Ring, δ(CN, CH_2)	1251	1277

Table S2. Assignment of experimental and DFT calculated characteristic IR bands for all isomers listed in figures 5B and S8B of M-PI for M=Li^+ and Na^+. Matching of the vibrational modes was achieved by visual inspection of the atomic displacements in internal coordinates.

		**ν(C=O) (cm^{-1})**		**ν Ar.(C=C) + ν (C-O^-) (cm^{-1})**	
		**Li-PI**	**Na-PI**	**Li-PI**	**Na-PI**
Exp.	-	*1637*	*1643*	*1536*	*1554*
	-	*1610*	*1602*	*1523*	*1525*
**i1**	-	*1682*	*1671*	1537	*1544*
**i2**	*Sym.*	*1703*	*1693*	*1588*	*1588*
	Asym.	*1660*	*1649*	*1523*	*1530*
**i3**	-	*1696*	*1685*	*1548*	*1548*
**i4**	-	*1685*	*1678*	*1541*	*1533*
**i5**	*Sym.*	*1682*	*1670*	*1548*	*1548*
	Asym.	*1656*	*1642*		
**i6**	*Sym.*	*1696*	*1685*	*1530*	*1544*
	Asym.	*1660*	*1645*		
**i7**	*Sym.*	*1696*	*1678*		
	Asym.	*1660*	*1641*		
**i8**	*Sym.*	*1699*	*1685*		
	Asym.	*1658*	*1645*		

Table S3. Relative energies (ΔE_{rel}) and Gibbs free energies of interaction (ΔG_{int}) between neutral NTCDI molecule and two Li or two Na atoms (bi-coordinated complexes), for all isomers listed in figures 5B and S8B.

Isomer	ΔE_{rel} [kJ/mol]		ΔG_{int} [kJ/mol]	
	2xLi-NTCDI	2xNa-NTCDI	2xLi-NTCDI	2xNa-NTCDI
i1	0	0	-346	-203
i2	3	1	-342	-202
i3	13	18	-331	-186
i4	15	26	-330	-178
i5	49	29	-296	-175
i6	54	36	-291	-168
i7	80	55	-265	-150
i8	92	54	-254	-151

Table S4. Enthalpies of formation (ΔH_{f1}) for the mono-coordinated 1xLi/1xNa-NTCDI (ΔH_{f1}) and bi-coordinated 2xLi/2xNa-NTDCI (ΔH_{f2}) complexes, and incremental enthalpies ($\Delta\Delta H_{b,2} = \Delta H_{f2} - \Delta H_{f1}$), indicative of cooperative stabilization.

Species	ΔH_{f1} [kJ/mol]	ΔH_{f2} [kJ/mol]	$\Delta\Delta H_{b,2}$ [kJ/mol]
Li complexes			-217.5
Na complexes	-134.0	-275.7	-141.7

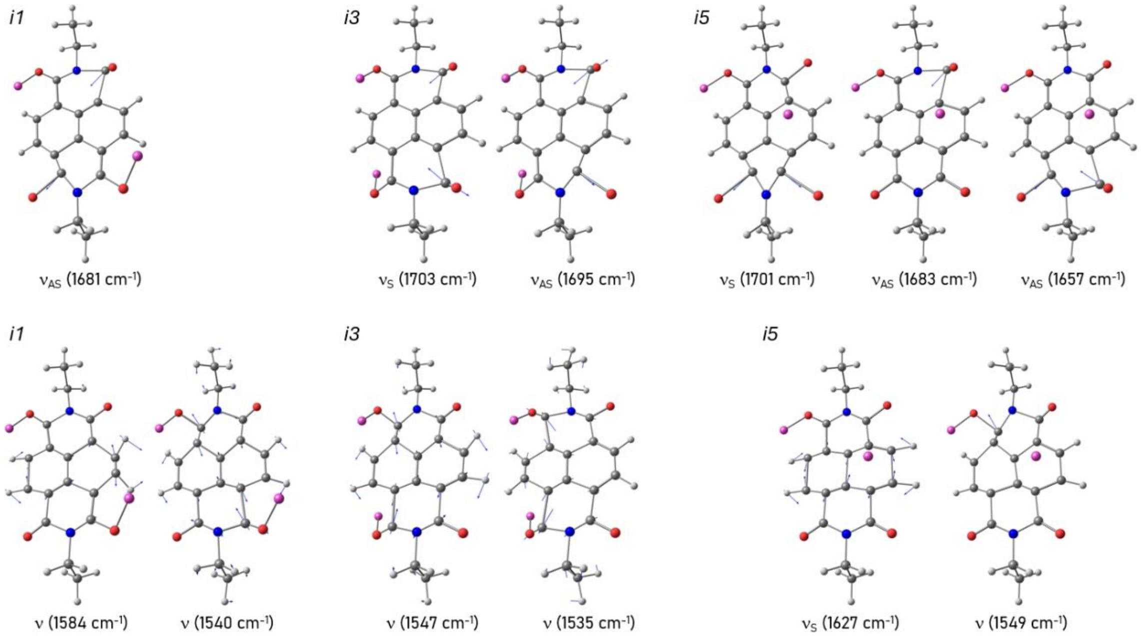

Figure S10 Illustration of the atomic displacements for selected IR bands, corresponding to vibrational modes of C-O bonds, and aromatic C-C and C-H bonds, of the isomers (i1), (i3) and (i5), presented in figure S8(B).

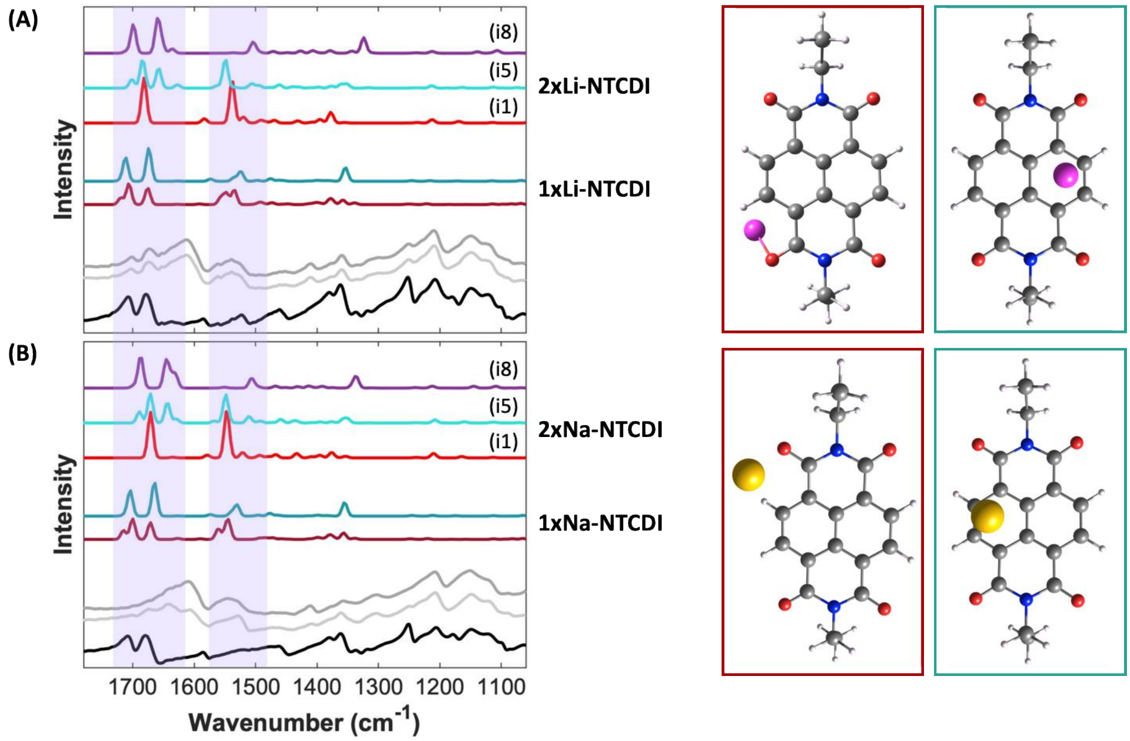

Figure S11. Experimental SR-FTIR spectrum of PI electrodes (pristine in black, at the end of first and second reduction in light grey and dark grey, respectively) *vs.* DFT calculated spectra for mono-coordinated 1xLi/1xNa-NTCDI complexes (in dark red and dark cyan) and for the bi-coordinated 2xLi/2xNa-NTCDI isomers (i1 in red, i5 in cyan, i8 in violet). Right panel shows representative optimized geometries of the mono-complexes. (A) in Li cell, and (B) in Na cell.

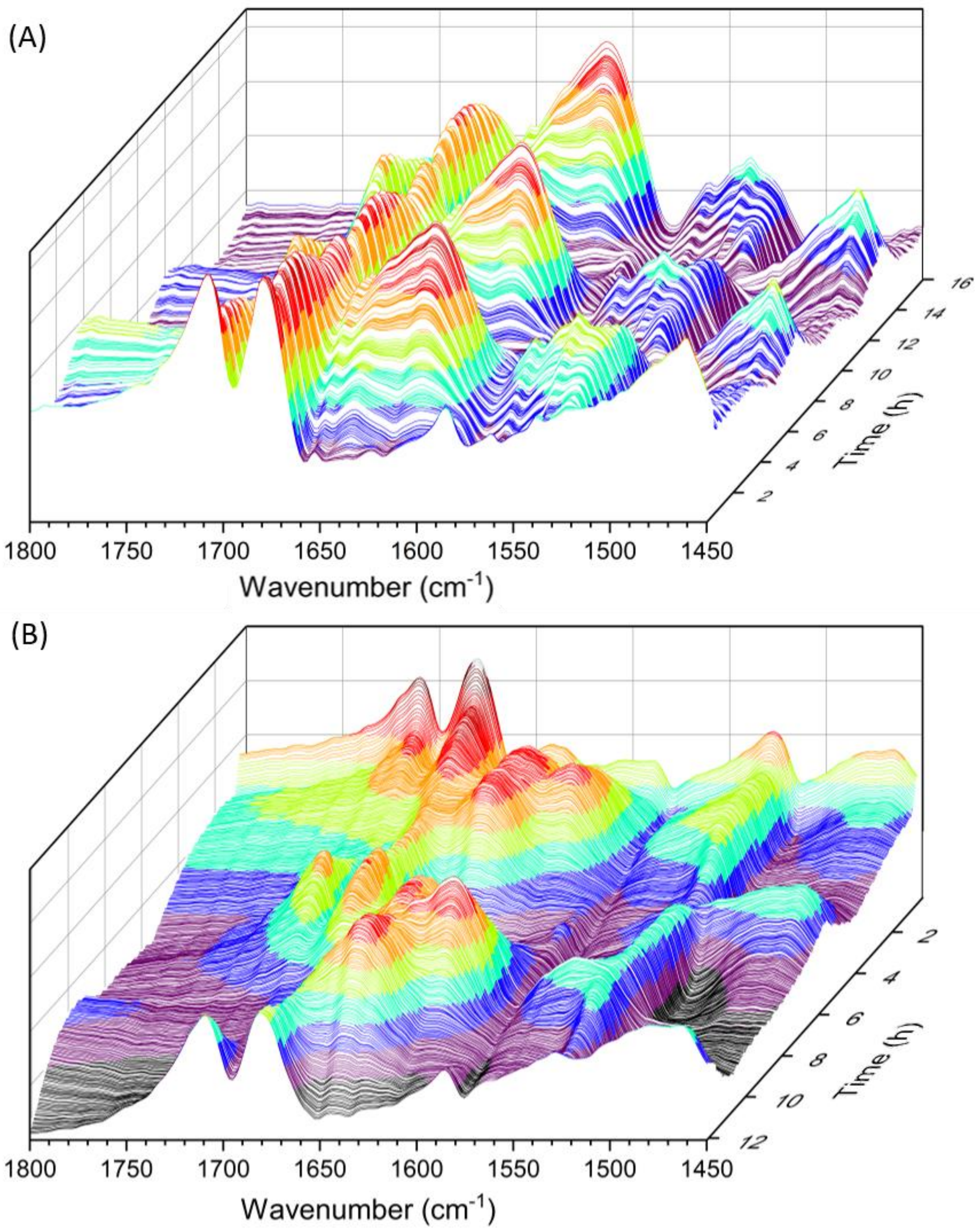

Figure. S12 3D plots of the operando SR-FTIR spectra of PI electrodes at C/4 in LiTFSI in DG (A) and in NaTFSI in DG (B).

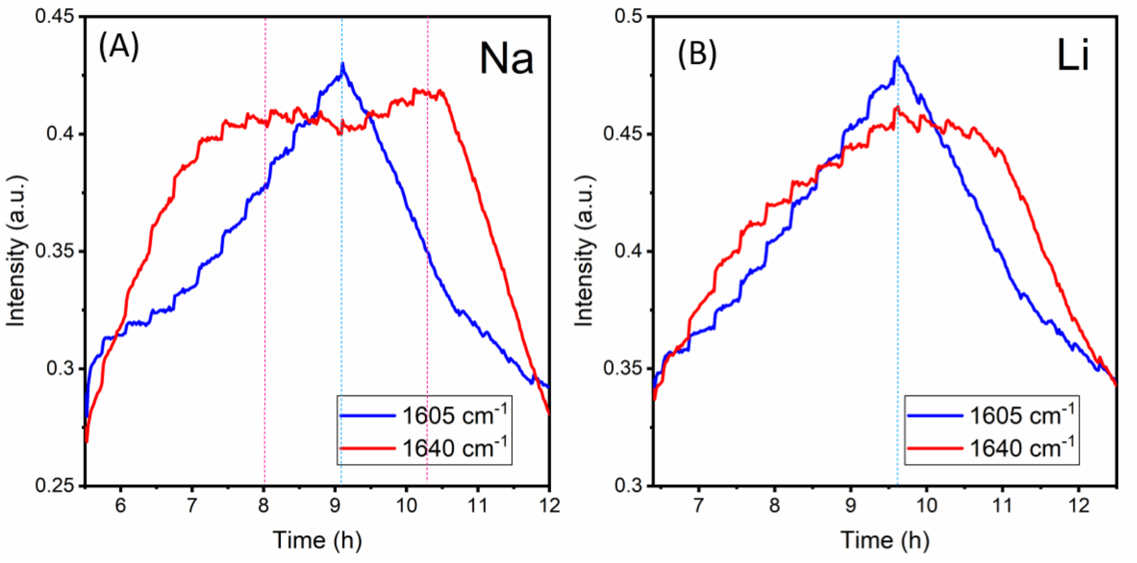

Figure. S13 intensity variation at 1605 and 1640 cm^{-1} of plots of the operando SR-FTIR spectra of PI electrodes at C/4 in NaTFSI in DG (A) and in LiTFSI in DG (B).

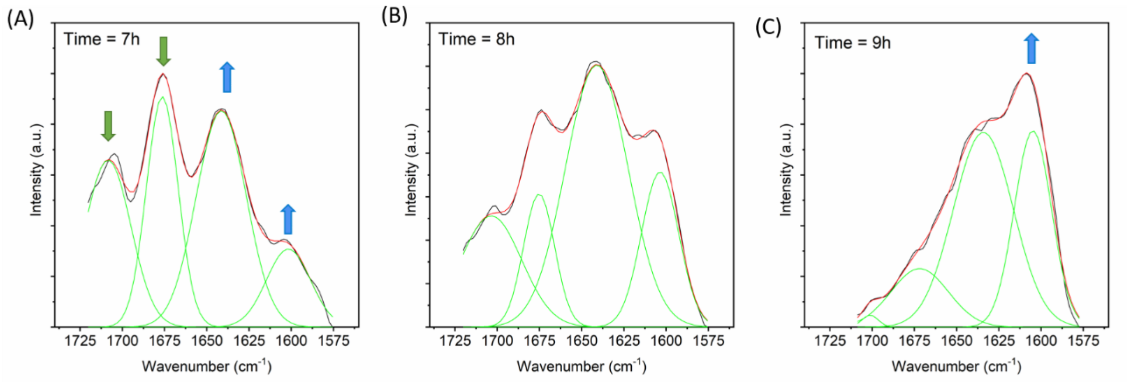

Figure. S14 Fitting with four components of operando SR-FTIR spectra of PI electrodes at C/4 in NaTFSI in DG at different states of charge 7h(A), 8h (B) and 9h (C).

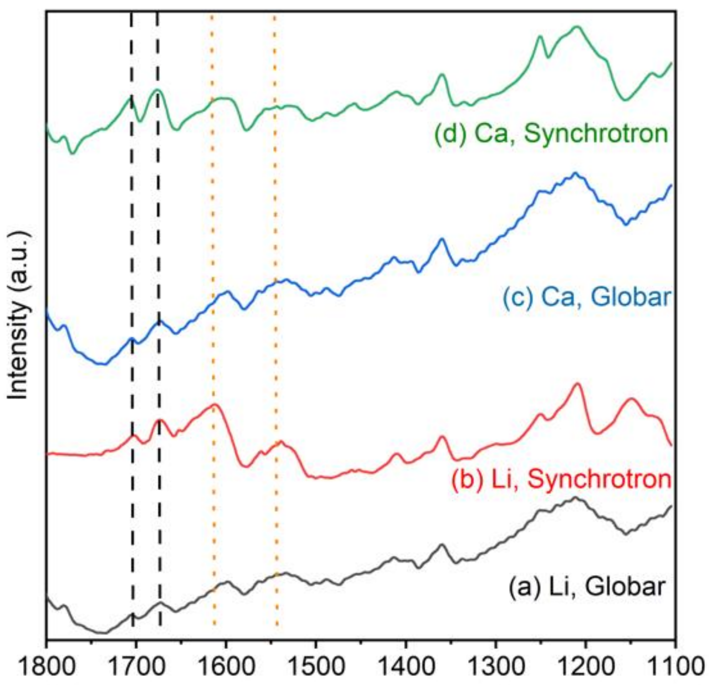

Figure. S15 Comparison of spectra's of PI electrodes measured at the final stage of reduction in Li and Ca cells using thermal internal source (Globar) and the synchrotron source.

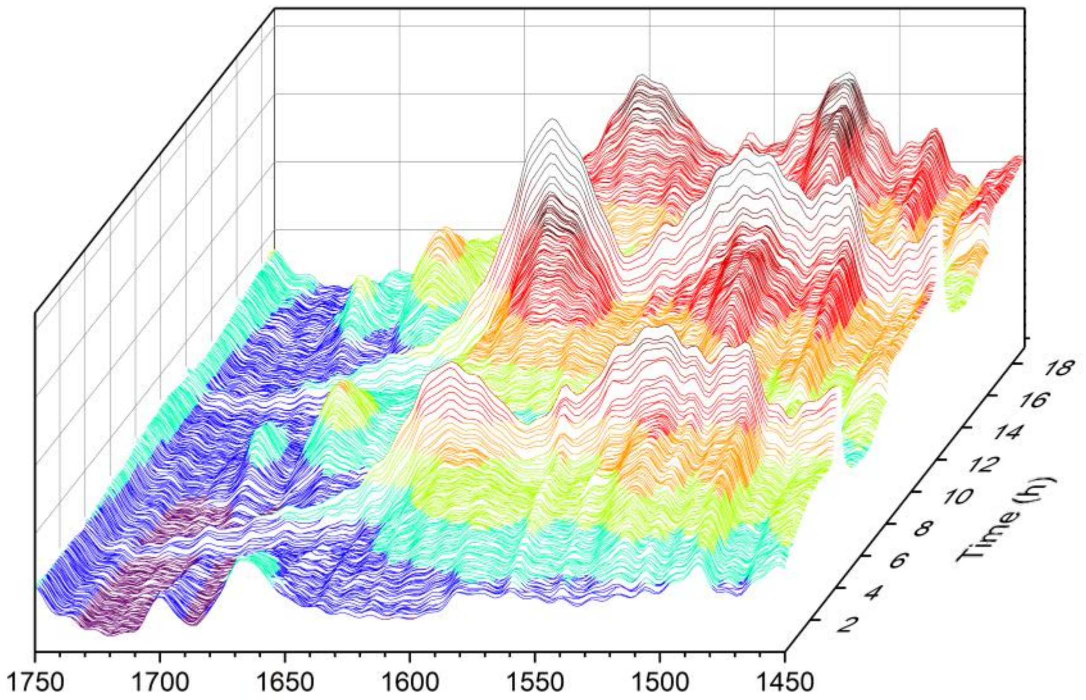

Figure. S16 3D plots of the operando FTIR spectra of PI electrodes in LiTFSI in EC:PC measured with the internal source (Globar) and a CaF_2 window.

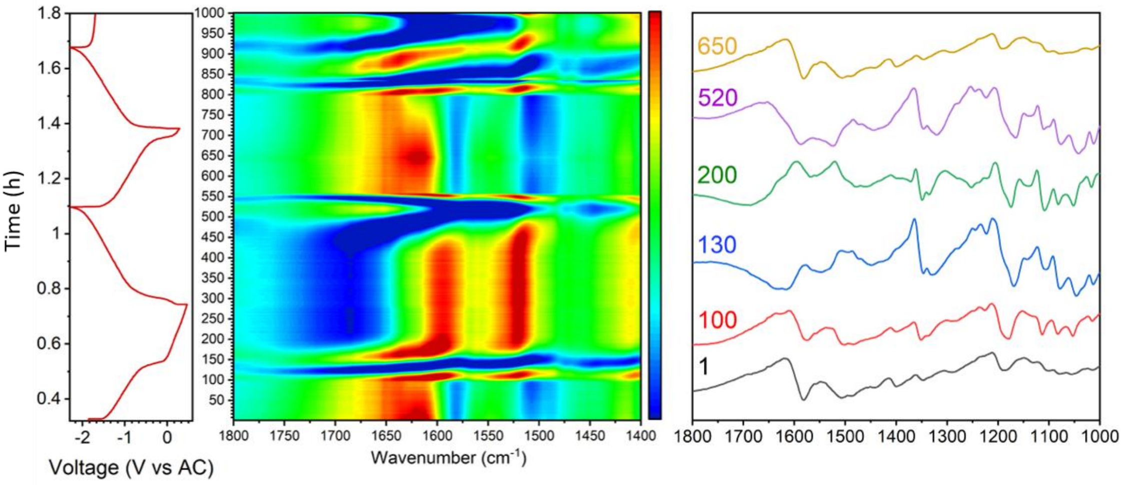

Figure. S17 The GCPL profile of PI at 2.5 C in LiTFSI in DG, its corresponding contour plot of the operando SR-FTIR spectra in the region 1400-1800 cm^{-1} and the selected spectra corresponding to different stages of charge. Vertical axis corresponds to spectrum number at specific cycling time.

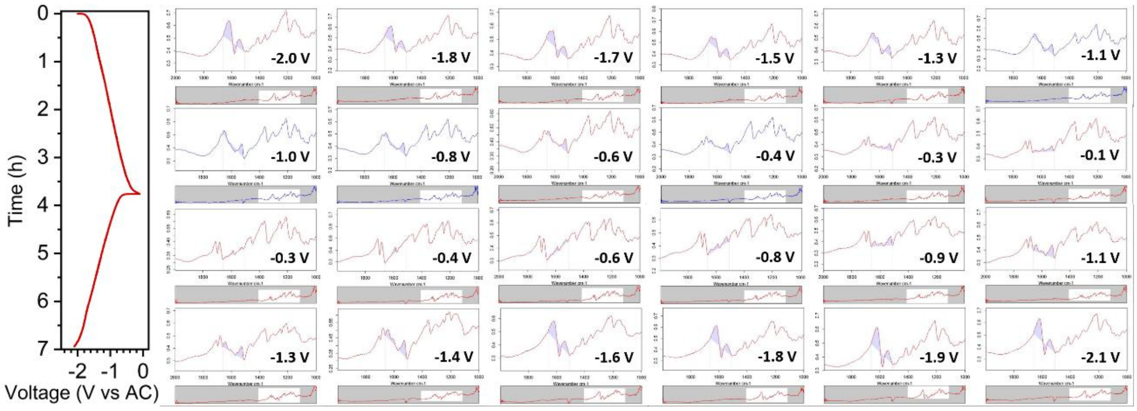

Figure. S18 GCPL profile of PI at C/4 in LiTFSI in DG (A) and a representative selection of 24 spectra of the 194 spectra that was measured at a single point of the 154 points of the corresponding microspectroscopy map. Showing the integrated intensity of the region (1508-1660 cm^{-1}) that corresponds to the two new bands formed upon ion coordination.

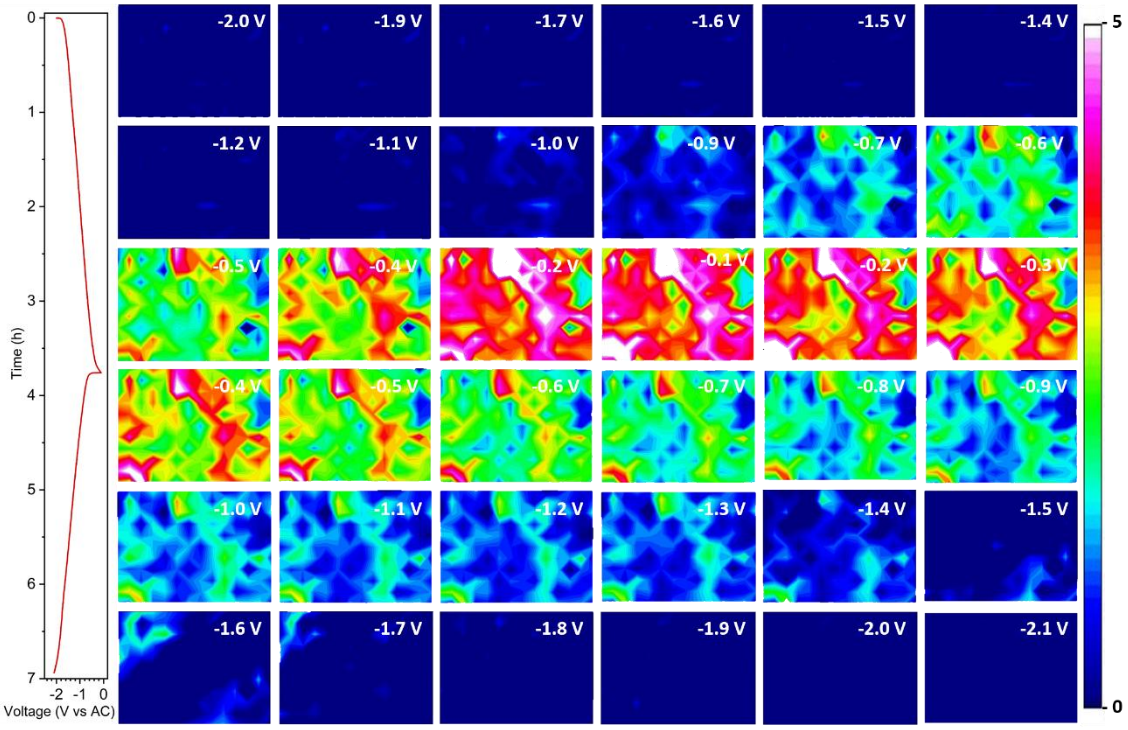

Figure. S19 GCPL profile of PI at C/4 in LiTFSI in DG and a representative selection of 36 microspectroscopy map (190 x 250 µm) of the 194 maps measured operando. The color maps represent the spatial distribution of the bands corresponding to the carbonyl groups, region 1662-1750 cm^{-1}.

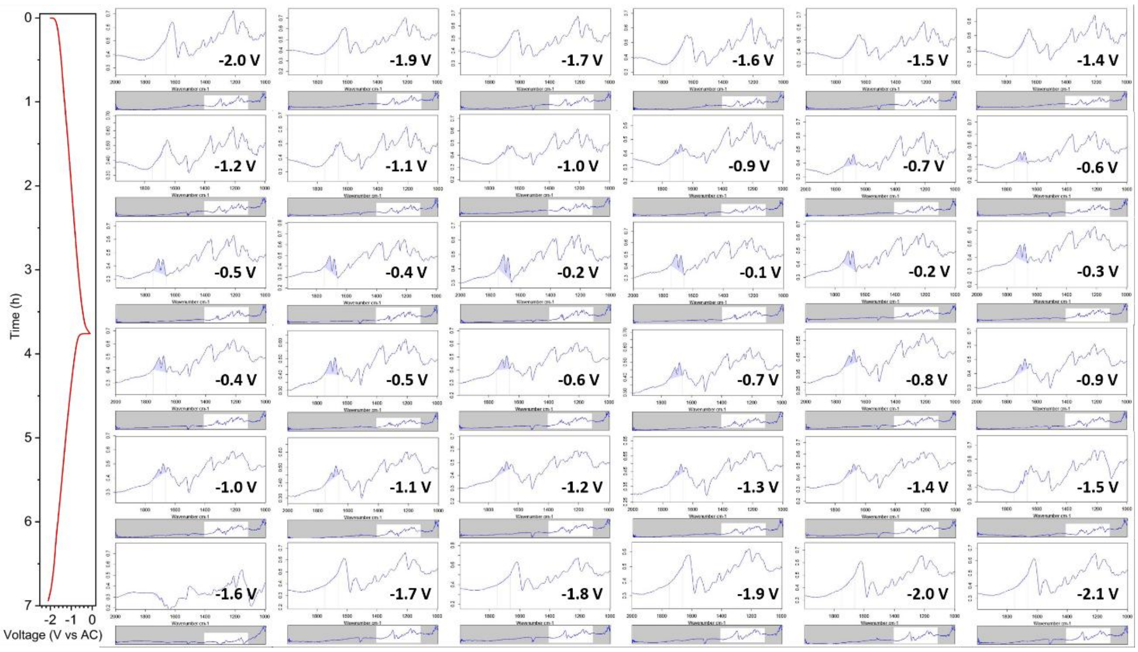

Figure. S20 The GCPL profile of PI at C/4 in LiTFSI in DG (A) and a representative selection of 36 spectra of the 194 spectra measured at a single point of the microspectroscopy map from Figure S15. Showing the integrated intensity of the carbonyl groups, region 1662-1750 cm^{-1}.

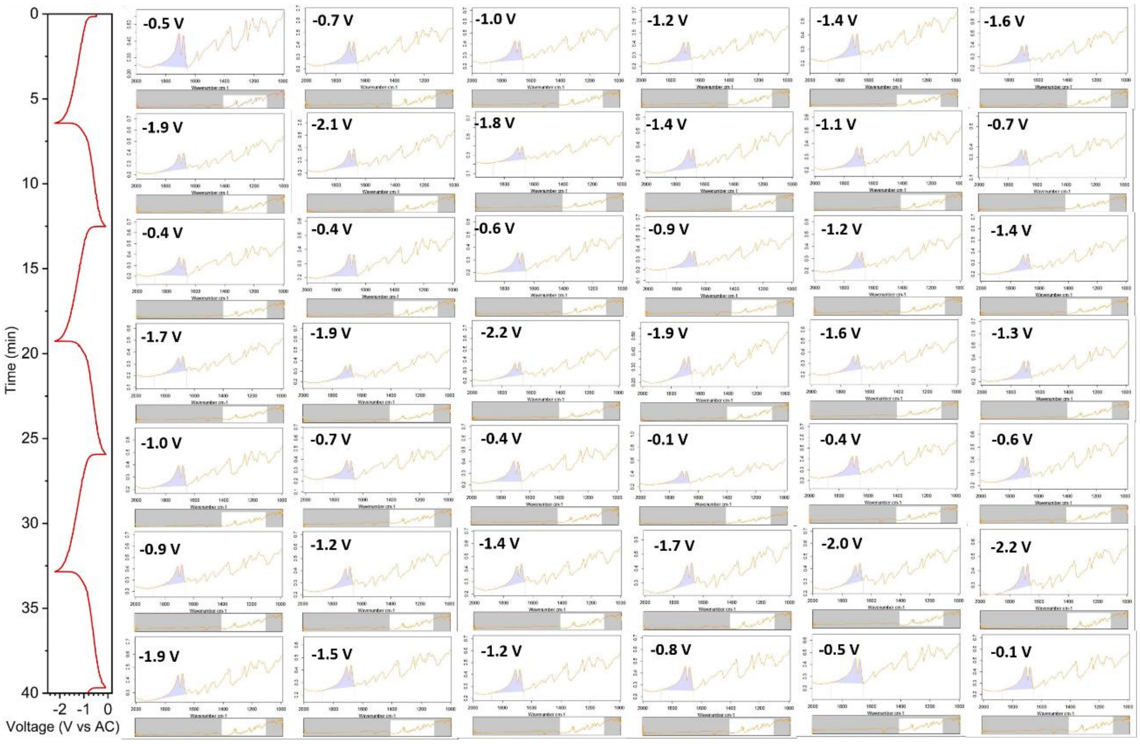

Figure. S21 The GCPL profile of PI at 2.5C in NaTFSI in DG (A) and 42 spectra corresponding to one single point of the microspectroscopy map from Figure S19. Showing the integrated intensity of the carbonyl groups, region 1660-1850 cm^{-1}.

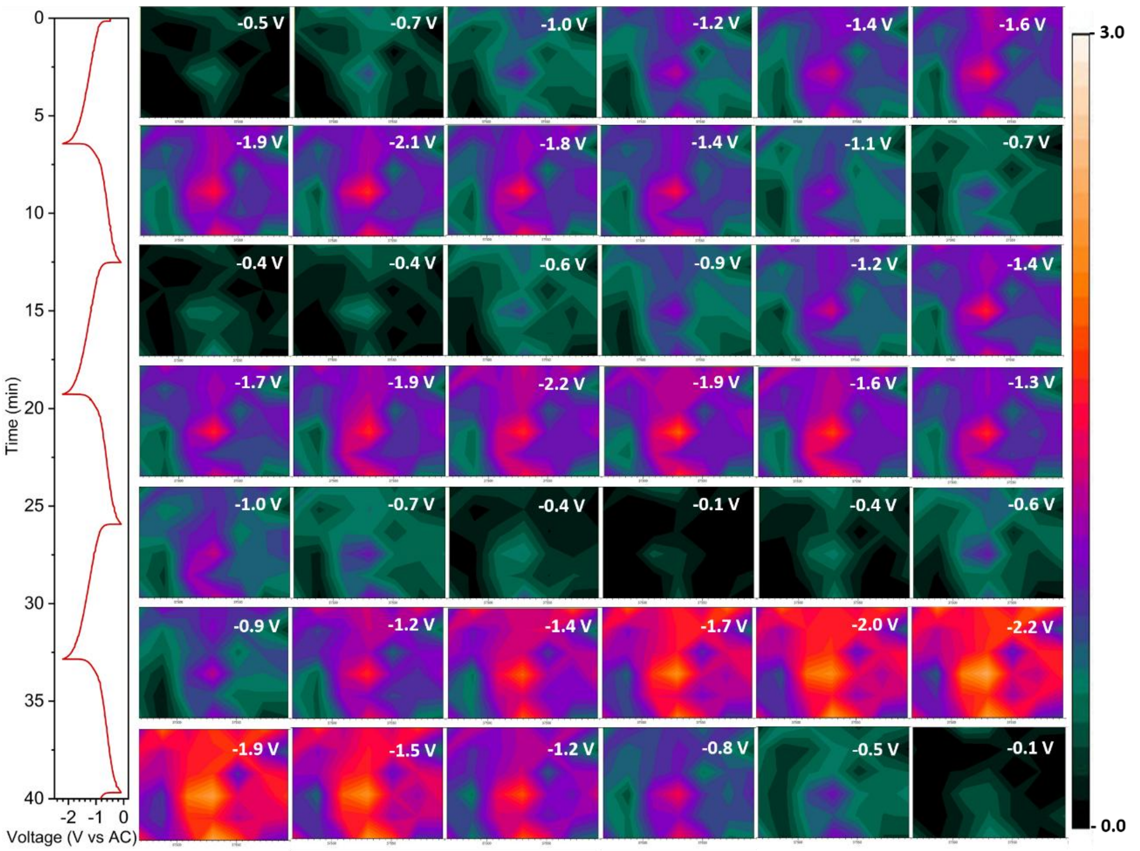

Figure. S22 The GCPL profile of PI at 2.5C in NaTFSI in DG and the corresponding operando SR-FTIR microspectroscopy maps of the electrode at different stages of charge. Color scale

correspond to the chemical distribution of the IR bands appearing in the region (1510-1570 cm^{-1}) in the scanned area of the electrode (150 x 95 μm).

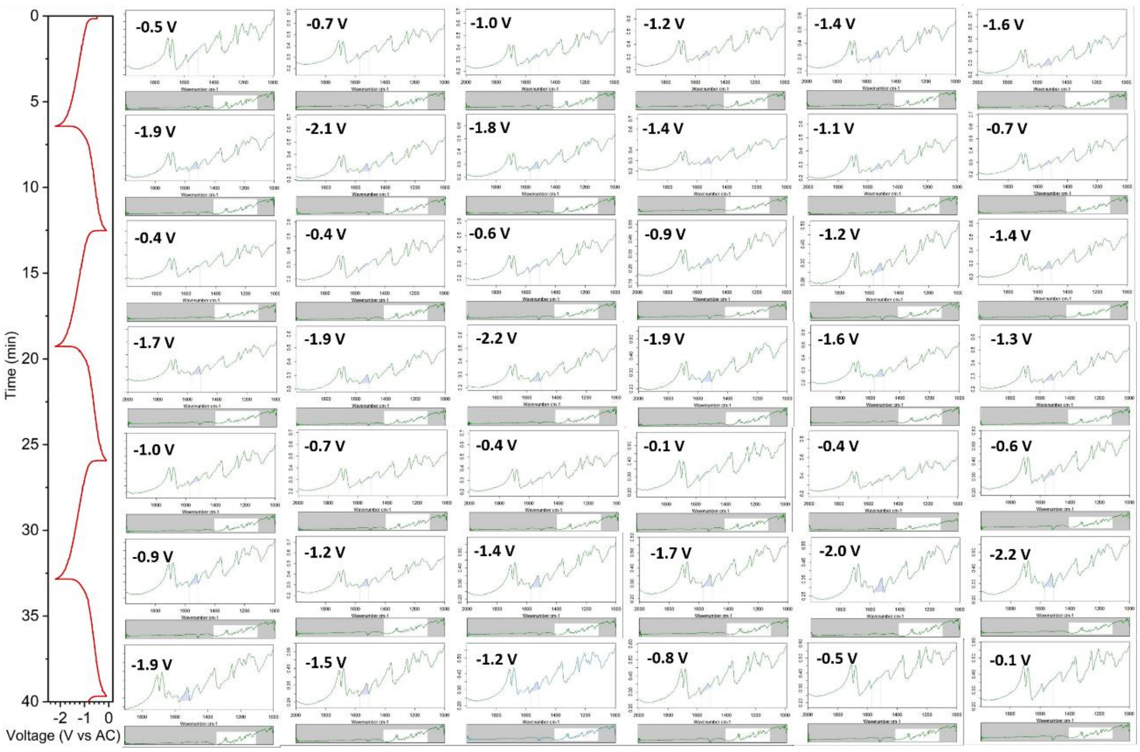

Figure. S23 The GCPL profile of PI at 2.5C in NaTFSI in DG (A) and 42 spectra corresponding to one single point of the microspectroscopy map. Showing the integrated intensity of the band appearing in the region 1510-1570 cm^{-1}.

References

1. Monti, D.; Patil, N.; Black, A. P.; Raptis, D.; Mavrandonakis, A.; Froudakis, G. E.; Yousef, I.; Goujon, N.; Mecerreyes, D.; Marcilla, R.; Ponrouch, A. Polyimides as Promising Cathodes for Metal-Organic Batteries: A Comparison between Divalent (Ca^{2+}, Mg^{2+}) and Monovalent (Li^{+}, Na^{+}) Cations. *ACS Appl. Energy Mater.* **2023**, *6* (13), 7250–7257. https://doi.org/10.1021/ACSAEM.3C00969/ASSET/IMAGES/LARGE/AE3C00969_0006.JPEG.
